# Effect of the Consumption of Species from the Zingiberaceae or Berberidaceae Family on Glycemic Profile Parameters: A Systematic Review and Meta-Analysis

**DOI:** 10.3390/ijms26125565

**Published:** 2025-06-10

**Authors:** Desirée Victoria-Montesinos, Begoña Cerdá Martínez-Pujalte, Pilar Zafrilla, Pura Ballester, Ana María García-Muñoz

**Affiliations:** Faculty of Pharmacy and Nutrition, UCAM Universidad Católica de Murcia, 30107 Murcia, Spain; dvictoria@ucam.edu (D.V.-M.); bcerda@ucam.edu (B.C.M.-P.); mpzafrilla@ucam.edu (P.Z.); pballester@ucam.edu (P.B.)

**Keywords:** type 2 diabetes, glycemic control, Zingiberaceae, Berberidaceae, fasting blood glucose

## Abstract

Type 2 diabetes mellitus (T2 DM) is a global health issue linked to high morbidity and mortality due to complications such as cardiovascular disease and nephropathy. Conventional treatments often have side effects and limited glycemic control, leading to interest in alternative therapies. Plants from the Zingiberaceae and Berberidaceae families, traditionally used for their anti-diabetic properties, have emerged as potential adjuncts. This meta-analysis evaluates and compares their efficacy in improving glycemic control in individuals with T2 DM. A systematic literature search, following PRISMA guidelines, was conducted in PubMed, Web of Science, SCOPUS, and Cochrane, identifying 1269 studies, of which 58 met inclusion criteria. Only randomized controlled trials assessing effects on fasting blood glucose (FBS), HbA1c, fasting insulin, and HOMA-IR were included. Study quality and risk of bias were assessed using Cochrane’s RoB 2.0 tool. The review is registered in PROS-PERO (CRD42024516261). The analysis showed significant reductions in FBS (−1.06; 95% CI: −1.42 to −0.71), HbA1c (−1.42; 95% CI: −2.64 to −0.19), and fasting insulin (−0.75; 95% CI: −1.13 to −0.38) among participants using plant extracts, with stronger effects observed for the Berberidaceae species. HOMA-IR also decreased, indicating enhanced insulin sensitivity. While Berberidaceae showed higher effect sizes, Zingiberaceae species provided more consistent outcomes. Further research with standardized protocols is needed to confirm these results.

## 1. Introduction

Type 2 diabetes mellitus (T2 DM) represents one of the most pressing global health challenges, with its prevalence rising at an alarming rate. As of 2019, approximately 463 million adults were living with diabetes, a number projected to increase to 700 million by 2045 [1]. T2 DM is primarily a metabolic disorder characterized by chronic hyperglycemia, which results from defects in insulin secretion, insulin action, or both. If not adequately managed, the disease often leads to severe complications such as cardiovascular disease, nephropathy, neuropathy, and retinopathy, contributing significantly to morbidity and mortality [2].

Standard treatments for T2 DM, including oral hypoglycemic agents such as metformin, sulfonylureas, and insulin, are effective in managing blood glucose levels. However, the long-term use of these medications is frequently associated with adverse side effects, including hypoglycemia, gastrointestinal distress, and weight gain [3]. Furthermore, despite the availability of these treatments, many patients struggle to achieve optimal glycemic control, underscoring the need for complementary and alternative therapeutic options that are safer and potentially more sustainable in the long term.

In recent years, interest has surged in the use of medicinal plants as adjunct therapies for T2 DM. These natural compounds, which have been used for centuries in traditional medicine, offer promising glycemic control with fewer side effects compared to conventional pharmacotherapy [4]. Notably, species from the Zingiberaceae and Berberidaceae families have drawn significant attention for their potential hypoglycemic properties. Plants from the Zingiberaceae family, such as *Curcuma longa* (turmeric), *Zingiber officinale* (ginger), and *Elettaria cardamomum* (cardamom), contain diverse bioactive compounds, including diarylheptanoids (e.g., curcumin, demethoxycurcumin), phenolics (e.g., gingerol, shogaol, zingerone), and terpenoids (e.g., zingiberene). These constituents exert antidiabetic effects through mechanisms such as antioxidant and anti-inflammatory action, the enhancement of glucose uptake via GLUT4, and the preservation of pancreatic β-cell function [5,6].

Similarly, the Berberidaceae family, which includes species such as *Berberis aristata* and *Berberis vulgaris*, typically contains a rich array of secondary metabolites, primarily isoquinoline alkaloids, along with flavonoids, phenolic acids, and, in some species, lignans. These plants are especially rich in berberine, palmatine, and berbamine. These compounds are known to activate AMP-activated protein kinase (AMPK), reduce hepatic gluconeogenesis, improve insulin signaling, and modulate gut microbiota composition [5,6]. Taken together, these plant families represent rich sources of phytochemicals with antidiabetic potential, particularly alkaloids, diarylheptanoids, flavonoids, terpenoids, and phenolic acids, which target key metabolic pathways involved in glycemic regulation [7].

Despite the wealth of preclinical and clinical evidence supporting the use of these plants in managing T2 DM, the outcomes from human studies remain inconsistent. Variability in study design, plant preparation, dosage, and duration of treatment contribute to these conflicting results, making it difficult to draw definitive conclusions about their effectiveness. Furthermore, most studies have focused on single plant species or isolated compounds, without comprehensive comparisons between plant families or across multiple glycemic control parameters [8]. As such, there is a need for a systematic and comprehensive evaluation of the clinical evidence to determine the true impact of these medicinal plants on glycemic control.

Several meta-analyses have investigated the effects of medicinal plants on metabolic health and glycemic control in individuals with T2 DM. For example, a 2022 meta-analysis by Zhang et al. assessed the impact of the Zingiberaceae species on cardiovascular risk factors in T2 DM patients, finding significant improvements in fasting blood glucose (FBG), HbA1c, insulin sensitivity, and lipid profiles [9]. Similarly, another recent meta-analysis focused on the anti-diabetic effects of the Zingiberaceae species reported significant reductions in glycemic markers and improvements in inflammatory profiles, suggesting that bioactive compounds like gingerol may have substantial therapeutic potential [10]. However, both analyses primarily concentrated on the cardiovascular and metabolic benefits of the Zingiberaceae species alone, without incorporating a direct comparison to other medicinal plant families.

In contrast, our study seeks to build upon this existing body of knowledge by including both the Zingiberaceae and Berberidaceae species in the analysis, allowing for a more comprehensive understanding of the relative efficacy of these two plant families on glycemic control. Unlike previous meta-analyses, which largely focused on individual species or a single plant family, this review systematically compares the effects of both the Zingiberaceae and Berberidaceae species on key parameters such as fasting blood glucose, HbA1c, fasting insulin, and HOMA-IR. This approach will enable us to identify whether there are significant differences in the hypoglycemic potential of these plant families, offering new insights into their comparative therapeutic value for managing T2 DM.

## 2. Materials and Methods

### 2.1. Literature Search

This meta-analysis was conducted in accordance with the guidelines outlined in the Preferred Reporting Items for Systematic Reviews and Meta-Analyses (PRISMA) [11]. The completed PRISMA checklist is provided in Figure S14. The protocol for this study was registered in the International Prospective Register of Systematic Reviews (PROSPERO CRD42024516261).

### 2.2. Eligibility Criteria

This review was conducted to address the question: What are the effects of the consumption of species of the family Zingiberaceae or of the family Berberidaceae on parameters of the glycemic profile?

Table 1 shows the inclusion and exclusion criteria used for this meta-analysis, structured according to the PICOS criteria.

Studies were included if they met the following criteria: (a) participants: individuals with alterations in the glycemic profile, such as those with prediabetes or type 2 diabetes mellitus; (b) outcomes: measurable changes in parameters related to the glycemic profile; (c) study design: randomized controlled trials with both an experimental group (receiving the intervention) and a control group. To minimize inconsistencies in quality assessment and data extraction, only studies published in English in peer-reviewed journals were included, due to limited access to reliable translation resources for non-English publications.

Exclusion criteria were as follows: (a) participants who did not have alterations in the glycemic profile; (b) studies that did not involve the consumption of species from the Zingiberaceae or Berberidaceae families or their extracted bioactives; (c) studies with insufficient information or where data extraction was not possible; (d) studies published in languages other than English; (e) studies that did not assess chronic consumption or lacked a control group; (f) studies that used a mixture of several components; (g) studies that were inaccessible for review or involved duplicate samples; (h) case–control studies, cohort analyses, individual case reports, collections of case studies, systematic reviews, meta-analyses, and experimental animal studies.

In this review, specific species from each family were selected for inclusion based on their documented effects on glycemic control and their prevalence in traditional medicinal practices. For the Zingiberaceae family, the species chosen were Turmeric (*Curcuma longa*), Ginger (*Zingiber officinale*), and Cardamom (*Elettaria cardamomum*). These species were selected due to their well-documented anti-inflammatory and hypoglycemic properties, which have been supported by both traditional use and scientific research. For the Berberidaceae family, the selected species included *Berberis aristata* (Indian Barberry), Berberis vulgaris (Common Barberry), *Coptis chinensis* (Huanglian), as well as *Mahonia aquifolium* (Oregon Grape) and *Epimedium* spp. These species were chosen primarily because they contain the bioactive compound berberine, which has shown significant potential in regulating blood glucose levels and improving insulin sensitivity.

### 2.3. Information Sources and Search Strategy

The systematic search was conducted in July 2024 by two independent researchers to identify relevant studies (A.M.G.-M. and D.V.-M.). The databases searched included PubMed, Web of Science, SCOPUS, and the Cochrane Database of Systematic Reviews. The search included all relevant studies published up to July 2024. The search strategy employed a combination of keywords and MeSH terms to thoroughly encompass studies related to the research question. For Zingiberaceae species, terms such as “Turmeric”, “*Curcuma longa*”, “Ginger”, “*Zingiber officinale*”, “Cardamom”, and “*Elettaria cardamomum*” were utilized. To identify studies involving Berberidaceae species, keywords included “*Berberis aristata*”, “*Indian Barberry*”, “*Berberis vulgaris*”, “*Common Barberry*”, “*Mahonia aquifolium*”, “*Oregon Grape*”, “*Epimedium* spp.”, “*Coptis chinensis*”, and “*Huanglian*”. Keywords related to glycemic profile parameters covered terms like “Diabetes Mellitus”, “Blood Glucose”, “Glycemic Control”, “Insulin”, “Insulin Sensitivity”, “HbA1c”, and other aspects of glucose metabolism. Additionally, terms such as “Clinical Trial”, “Randomized Controlled Trial”, “Control Group”, and “Placebo” were included to focus on specific study designs.

All search terms were tailored to fit the specific requirements and filters of each database. Additionally, the reference lists of each included article were thoroughly reviewed to ensure that no eligible studies were overlooked.

### 2.4. Selection Process

After conducting the search across the different databases, Zotero software (version 6.0.36, Corporation for Digital Scholarship, Vienna, VA, USA) was used to remove duplicate entries. Two members of the research team, A.M.G.-M. and D.V.-M., independently reviewed the titles and abstracts to carry out the selection process, identifying relevant articles for further screening. Any disagreements during the selection process were resolved by a third researcher (B.C.M.-P.).

### 2.5. Data Items

For data extraction, the initial phase was conducted by two authors, A.M.G.-M. and B.C.M.-P., followed by confirmation of the extracted data by two additional authors, D.V.-M. and P.Z. The PICOS strategy [12] was employed to gather demographic details of the included populations and to characterize each intervention. Information extracted included the country of origin, study design, publication date, daily dosage, consumption period, participants’ health conditions, and the variables measured in each study. The studies were categorized based on their investigation of the effects of the Zingiberaceae and Berberidaceae species on glycemic profile parameters. Additional data, such as changes in FBS, HbA1c levels, fasting insulin levels, HOMA-IR index, 2hPG, and other glycemic control markers, such as body weight and BMI, were also recorded.

### 2.6. Quality Assessment

The risk of bias was assessed using the Cochrane Collaboration’s Risk of Bias (RoB 2.0) tool [13]. This comprehensive tool evaluates five key domains: (1) bias arising from the randomization process, (2) bias due to deviations from intended interventions, (3) bias due to missing outcome data, (4) bias in measurement of the outcome, and (5) bias in selection of the reported result. Each domain is assessed to determine the level of bias, which is then classified as low risk, high risk, or some concerns. A low-risk rating indicates that bias is unlikely to impact the study results, while a high-risk rating suggests a significant potential to affect the outcomes. The “some concerns” category is used when there is insufficient information to make a definitive judgment about the risk of bias.

The risk of bias was independently assessed by two reviewers, D.V.-M. and A.M.G.-M. In instances where discrepancies arose, a third reviewer, P.Z., was consulted to reach a consensus.

### 2.7. Synthesis Methods

The primary effect size of the interventions on glycemic profile parameters was quantified using the standardized mean difference (SMD), incorporating Hedges’ g to adjust for small sample sizes. Meta-analyses were conducted using a random-effects model with the Restricted Maximum Likelihood (REML) method to calculate the pooled estimate of effect size and 95% confidence intervals (CIs). Standard deviations were derived from standard errors or confidence intervals as necessary. All data transformations were performed following the guidelines outlined in the Cochrane Handbook [14]. When necessary, data were extracted from graphs using WebPlotDigitizer (version 4.5, Ankit Rohatgi, 2021 [15]), with accuracy ensured by independent reviewers who performed double-checks.

In addition to the primary analyses, subgroup analyses will be performed to compare the efficacy of species from the Zingiberaceae and Berberidaceae families. This approach will allow us to determine whether the therapeutic effects on parameters related to the glycemic profile differ significantly between the two plant families, providing a deeper insight into the relative efficacy of these bioactive compounds. Sensitivity analyses will be performed to ensure the robustness of the results, focusing on studies with a moderate–low risk of biased results, eliminating all studies with a high risk of bias. Additionally, to explore potential sources of heterogeneity among studies, a meta-regression analysis was conducted. The analysis assessed whether the dosage of the extract (g/day) and the duration of the intervention (weeks) were associated with the effect sizes of each outcome variable.

Forest plots were generated to visually represent the results, including 95% confidence intervals (CIs). The effect size of each study was calculated and categorized as small (0–0.20), medium (>0.20 to 0.50), or large (>0.50) to facilitate interpretation.

Heterogeneity among studies was assessed using the *I*^2^ statistic. *I*^2^ values were interpreted as follows: not significant (<40%), moderate (40–60%), substantial (60–75%), and considerable (75–100%). These metrics helped to understand the variability in effect sizes across studies and the consistency of the intervention effects.

To assess potential reporting biases, including publication bias and small-study effects, Egger’s test and funnel plots were used. A *p*-value of 0.05 was considered to be statistically significant.

All statistical analyses were conducted using Stata software (version 16.1; StataCorp, College Station, TX, USA), with a significance level set at *p* < 0.05.

## 3. Results

**Figure 1:**
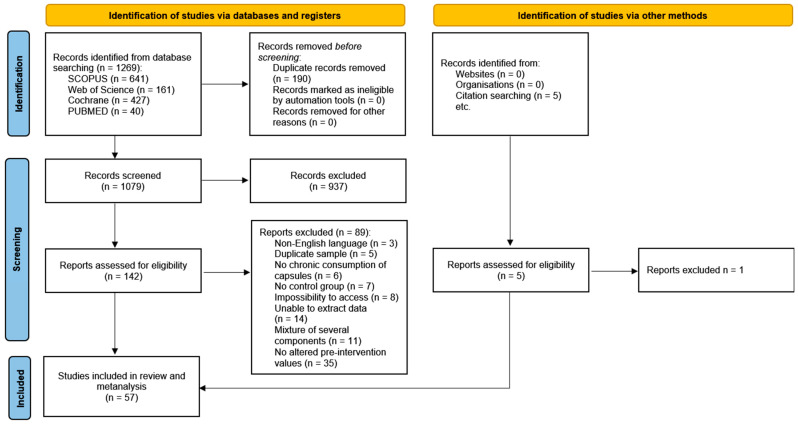
Flow chart.

A total of 1269 studies were initially identified across the four databases, with an additional 7 studies included from the references of other articles. After removing 190 duplicates, 1086 unique studies remained. Following the screening of titles and abstracts, 937 studies were excluded, leaving 147 articles to be assessed for eligibility. After a thorough review, 90 articles were excluded for various reasons, which are detailed in Figure 1. Finally, 58 studies were deemed eligible and included in the meta-analysis (Figure 1) [5,16,17,18,19,20,21,22,23,24,25,26,27,28,29,30,31,32,33,34,35,36,37,38,39,40,41,42,43,44,45,46,47,48,49,50,51,52,53,54,55,56,57,58,59,60,61,62,63,64,65,66,67,68,69,70,71,72,73].

### 3.1. Study Characteristics

The main characteristics of the 58 included studies are summarized in Table 2. The articles were published between 2014 and 2023. A total of 4354 participants (59.5% women) with a mean age of 50.5 ± 9.7 years were included in the present meta-analysis. Regarding BMI, the mean value across studies was 28.5 ± 2.3 kg/m^2^. In terms of geographical distribution, the studies represented nine different countries: the United States [57], China [5,27,31,38,45], India [22,33,54,59,67], Iran [16,17,19,20,21,23,24,25,29,32,36,37,39,41,42,43,44,46,47,48,50,51,52,53,55,58,60,61,62,63,64,68,69,70,72,73], Pakistan [18], Brazil [28,56,71], Italy [30,34,40,65,66], Thailand [26], and Egypt [40].

In 57 studies, FBS was measured before and after the intervention [5,16,17,18,19,20,21,22,23,24,25,26,27,28,29,30,31,32,33,34,35,36,37,38,39,41,42,43,44,45,46,47,48,50,51,52,53,54,55,56,57,58,59,60,61,62,63,64,65,66,67,68,69,70,71,72,73], while 38 studies reported HbA1c levels [5,16,17,19,20,21,22,23,25,28,29,31,34,35,37,38,39,40,42,43,45,46,48,50,52,53,54,55,56,58,59,61,63,65,68,69,71,73]. Fasting insulin levels and HOMA-IR index were evaluated in 31 [5,16,17,19,23,25,29,30,33,35,36,41,42,45,46,50,52,54,55,56,58,59,60,64,65,66,68,69,70,71,73] and 32 studies [5,16,17,19,25,27,28,29,30,33,35,40,41,42,45,50,52,54,56,58,59,60,62,64,65,68,69,70,71,73], respectively. Additionally, 16 studies measured other related parameters such as 2hPG [5,21,22,27,34,35,37,38,39,45,46,47,54,59,64,72]. Body weight and BMI changes were reported in 27 [5,16,19,20,21,23,24,25,29,32,33,34,41,42,44,46,50,52,54,57,58,60,62,63,68,69,71] and 36 studies [5,16,17,19,20,21,23,24,25,29,30,32,33,34,35,38,41,42,44,46,50,52,53,54,56,58,60,61,62,63,64,66,68,69,71,73], respectively.

Regarding the extract used, a total of 40 studies focused on species from the Zingiberaceae family, while 18 studies investigated species from the Berberidaceae family. The average daily dosage for the Zingiberaceae species was 1.1 ± 2.4 g, and for the Berberidaceae species, it was 2.0 ± 2.6 g. The average duration of intervention was 10.1 ± 2.5 weeks for the Zingiberaceae species and 24.0 ± 19.4 weeks for the Berberidaceae species. The studies included a variety of health conditions among participants, predominantly focusing on T2 DM, with 33 studies.

### 3.2. Bioactive Compound Supplementation

All the studies included in this meta-analysis implemented supplementation in the experimental groups, focusing on extracts from the Zingiberaceae and Berberidaceae species. Several studies used *Curcuma longa* (turmeric) and *Zingiber officinale* (ginger) as the primary bioactive compounds. For example, some studies provided turmeric in various forms, such as standardized extracts or nano-formulations, with daily dosages ranging from 0.5 to 2.1 g. These turmeric supplements were often administered over periods ranging from 4 to 12 weeks. Green cardamom (*Elettaria cardamomum*) was also utilized in some studies, typically at a dosage of 3 g per day over 8 to 16 weeks.

For the Berberidaceae family, the supplementation predominantly featured berberine, either alone or in combination with other compounds like fenugreek. Dosages of berberine varied from 0.3 to 10 g per day, with study durations extending up to 96 weeks.

### 3.3. Risk of Study Bias

**Figure 2:**
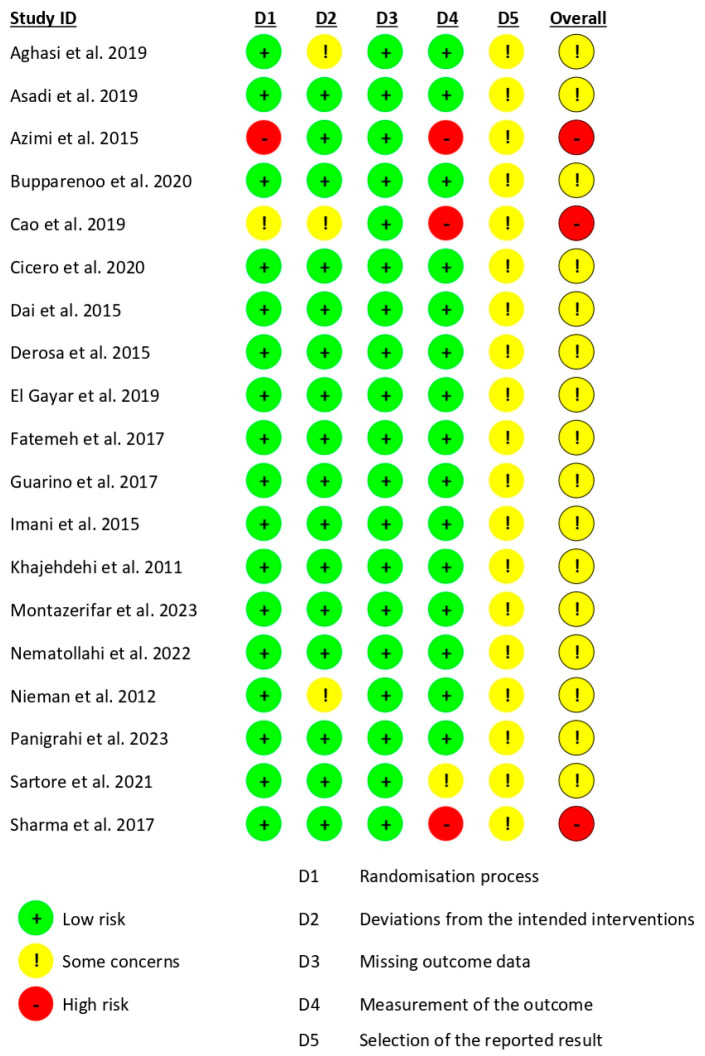
Graph of RoB 2.0 for each study based on the five domains defined by Cochrane, specifically for per-protocol studies [17,21,23,26,27,30,31,34,35,36,40,44,47,52,55,57,59,66,67].

**Figure 3:**
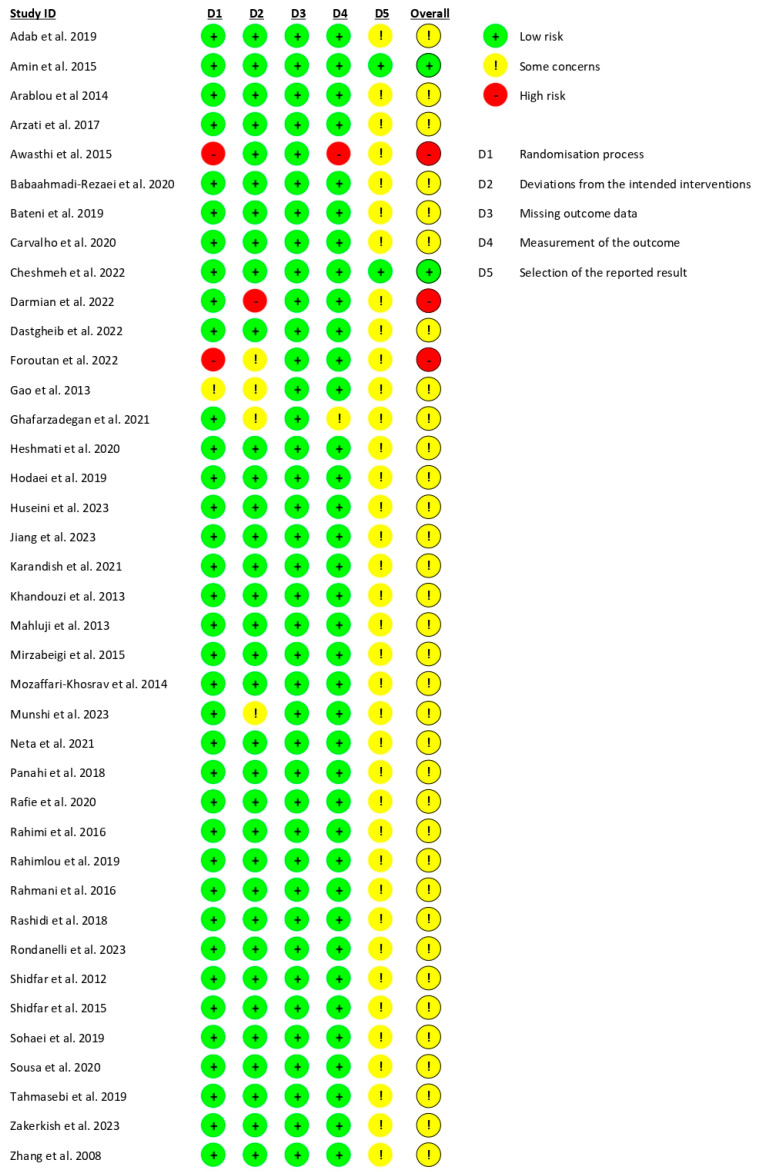
Graph of RoB 2.0 for each study based on the five domains defined by Cochrane, specifically for intention-to-treat studies [5,16,18,19,20,22,24,25,28,29,32,33,37,38,39,41,42,43,45,46,50,51,53,54,56,58,60,61,62,63,64,65,68,69,70,71,72,73].

The risk of bias was assessed using the Cochrane RoB 2.0 tool. The data from the studies included in this meta-analysis are presented in Figure 2 and Figure 3, according to the population of each study (per-protocol studies: 39; intention-to-treat studies: 19). Most of the studies presented “some concerns” regarding the risk of bias, particularly related to the D5 domain, which addresses the “selection of the reported result”. Furthermore, seven studies were identified as having a high risk of bias, indicating notable issues in their methodology or reporting practices.

### 3.4. Findings from Meta-Analysis

#### 3.4.1. FBS

The impact of bioactive compounds from the Zingiberaceae and Berberidaceae families on FBS levels has been a primary focus of numerous clinical studies, particularly in the context of T2 DM. This meta-analysis includes data from 57 studies, which collectively provide robust evidence of the efficacy of these compounds in reducing FBS [5,21,22,23,24,25,26,27,28,29,30,31,32,33,34,35,36,37,38,39,40,41,42,43,44,46,47,48,49,50,51,52,53,55,56,57,58,59,60,61,62,63,64,65,66,67,68,69,70,71,72,73,74,75,76,77].

**Figure 4:**
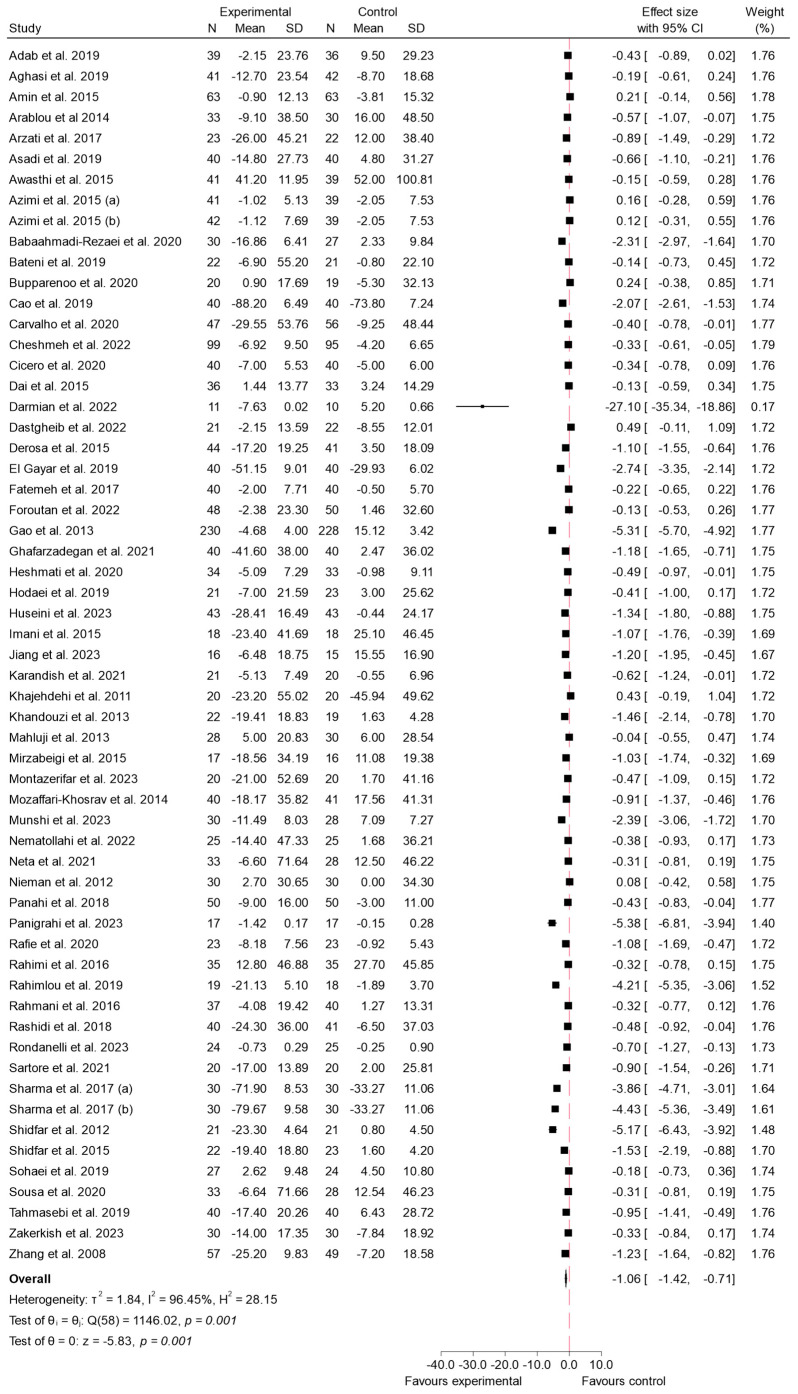
Comparisons of the effects of consumption of the Zingiberaceae and Berberidaceae species versus a control on FBS [5,16,17,18,19,20,21,22,23,24,25,26,27,28,29,30,31,32,33,34,35,36,37,38,39,40,41,42,43,44,45,46,47,48,49,50,51,52,53,54,55,56,57,58,59,60,61,62,63,64,65,66,67,68,69,70,71,72,73].

Overall, the combined effect size significantly favored the experimental group, with a global effect size of −1.06 (95% CI: −1.42 to −0.71; *p* < 0.001). This indicates that the consumption of these species led to a greater reduction in FBS levels compared to the control. However, there was substantial heterogeneity among the studies (*I*^2^ = 98.45%), suggesting considerable variability in individual study outcomes. Despite this, the overall analysis consistently demonstrated a significant reduction in FBS levels in the experimental group, underscoring the potential of the Zingiberaceae and Berberidaceae species in managing FBS. These results can be seen in Figure 4. To investigate this variability, a meta-regression analysis was performed to explore the influence of intervention duration and daily dosage on the variability of the effect sizes. A significant inverse association was observed between the daily dose of the plant extract and the magnitude of the effect on FBS levels (β = −0.275; *p* < 0.003), indicating that higher doses were associated with greater reductions in FBS. In contrast, intervention duration did not show a statistically significant association with the outcome (β = −0.8204; *p* = 0.110). The model accounted for approximately 19.76% of the heterogeneity.

Sensitivity analysis (Figure S1 of the Supplementary Materials) showed that after exclusion of 6 studies at high risk of bias [22,23,27,32,37,67], the pooled effect size was −0.96 (95% CI: −1.30 to −0.61; *I*^2^ = 95.63%). A funnel plot was used to visually assess potential asymmetry and publication bias in the included studies. The plot exhibited clear asymmetry, with some studies showing a significant deviation to the left and a clustering of points on the right side. In addition, Egger’s regression test was performed to quantify the presence of small-study effects. The results indicated a beta coefficient of β = −7.74 with a beta standard error of 0.86, with a z-value of −8.97 and a *p*-value of *p* < 0.001. These findings provide strong evidence of small-study effects and suggest significant publication bias in the meta-analysis. These results are displayed in Figure S2A. To further investigate this asymmetry, Egger’s test and Funnel plot were performed for each of the families. Analysis by family did not show contrasting levels of publication bias between studies (*p*-value < 0.001 in both cases; Figure S2B,C).

**Figure 5:**
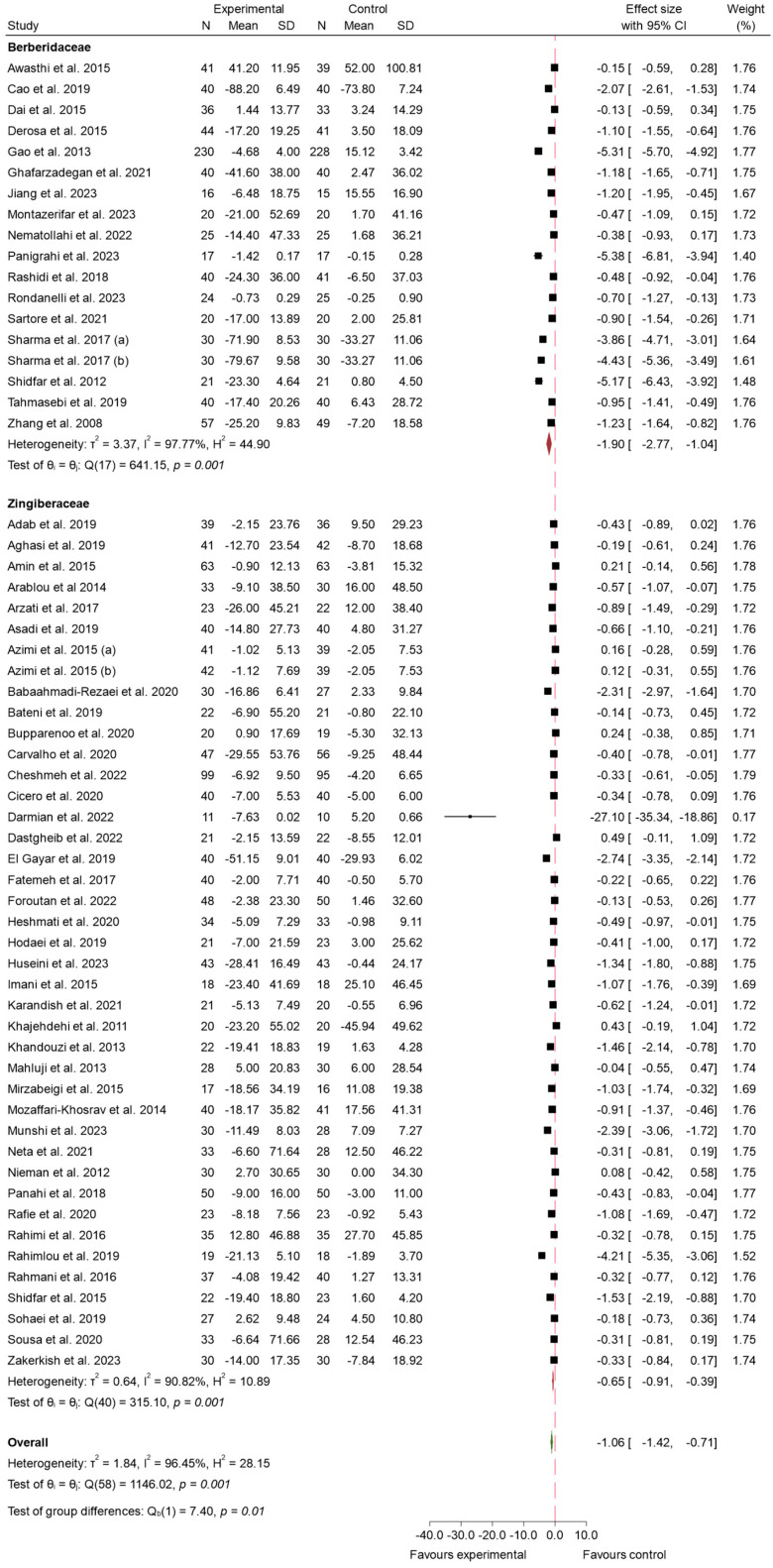
Subgroup analysis in relation to the family group for FBS [5,16,17,18,19,20,21,22,23,24,25,26,27,28,29,30,31,32,33,34,35,36,37,38,39,40,41,42,43,44,45,46,47,48,49,50,51,52,53,54,55,56,57,58,59,60,61,62,63,64,65,66,67,68,69,70,71,72,73].

To further investigate the heterogeneity observed in the meta-analysis, a subgroup analysis was conducted, which aimed to provide a deeper insight into the factors contributing to the variability of results between studies. This analysis stratified the studies according to the species consumed, specifically the Zingiberaceae and Berberidaceae families, to determine whether species type influenced the overall effect. Upon conducting the subgroup analysis by family, the studies examining the Berberidaceae species demonstrated a more pronounced effect compared to those investigating Zingiberaceae. The pooled effect size for Berberidaceae was −1.90 (95% CI: −2.77 to −1.04; *p* < 0.001), indicating a greater reduction in the evaluated outcomes. However, the heterogeneity among studies in this subgroup was high (*I*^2^ = 97.77%), suggesting substantial variability in individual results. In contrast, the Zingiberaceae species also showed a significant effect, with a pooled effect size of −0.65 (95% CI: −0.91 to −0.39; *p* < 0.001), though this effect was smaller compared to Berberidaceae. The heterogeneity in this subgroup was lower (*I*^2^ = 90.82%), indicating less variability across studies. The subgroup comparison is shown in Figure 5. The difference between groups was *p* < 0.01.

#### 3.4.2. HbA1c

**Figure 6:**
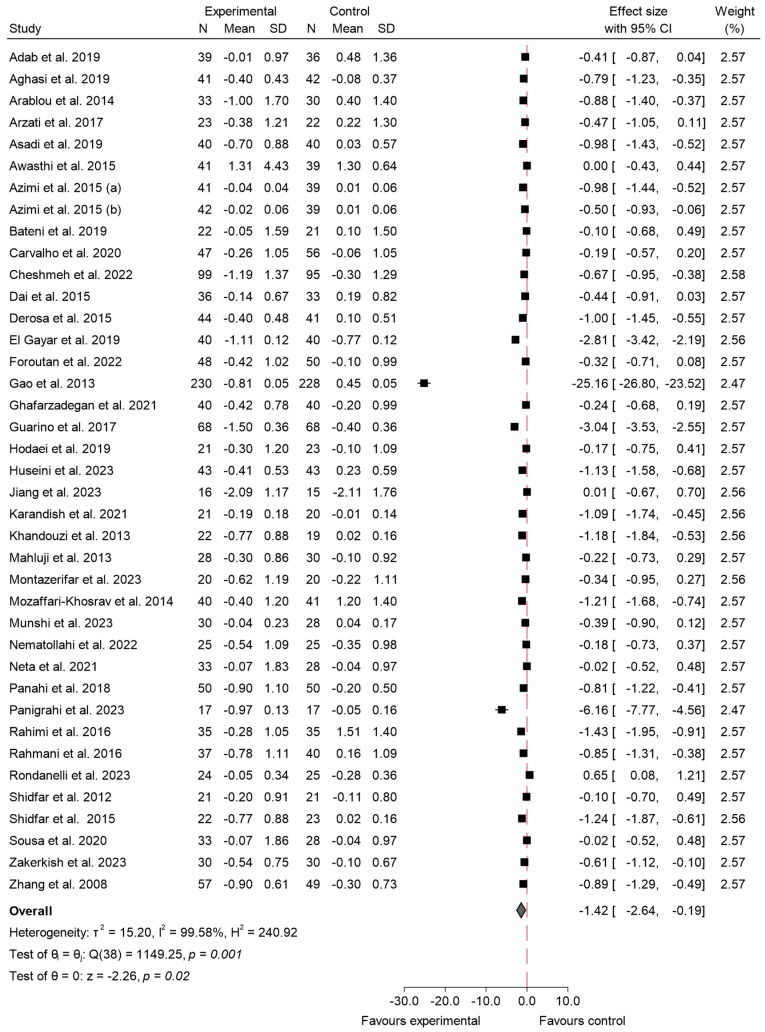
Comparisons of the effects of consumption of Zingiberaceae and Berberidaceae species versus a control on HbA1c [5,16,17,19,20,21,22,23,25,28,29,31,34,35,37,38,39,42,43,45,46,48,50,52,53,54,55,56,58,59,61,63,65,68,69,71,73].

In relation to the variable HbA1c, 38 studies assessed the effect of species from the Zingiberaceae and Berberidaceae families on this outcome [5,16,17,19,20,21,22,23,25,28,29,31,34,35,37,38,39,40,42,43,45,46,48,50,52,53,54,55,56,58,59,61,63,65,68,69,71,73]. The meta-analysis revealed a significant reduction in the experimental group compared to the control. The overall effect size was −1.42 (95% CI: −2.64 to −0.19; *p* < 0.02), indicating a marked decrease in HbA1c levels following the consumption of these species. The included studies exhibited a high degree of heterogeneity, with an *I*^2^ value of 99.58%, indicating substantial variability among the results. The findings from this analysis are presented in Figure 6. For HbA1c, meta-regression revealed a significant inverse association between daily dose and effect size (β = −1.07; *p* < 0.001), indicating that higher doses were associated with greater reductions in HbA1c levels. The duration of the intervention did not show a statistically significant effect (β = −0.05; *p* = 0.091). The regression model accounted for approximately 36.5% of the observed heterogeneity.

Sensitivity analysis (Figure S3) was conducted by excluding three studies with a high risk of bias [22,23,37]. Following this exclusion, the overall effect size slightly changed to −1.53 (95% CI: −2.90 to −0.16; *p* < 0.03), indicating a maintained but slightly stronger reduction in HbA1c levels compared to the initial analysis. Heterogeneity remained high, with an *I*^2^ of 99.61%, showing minimal improvement in consistency among the studies. The corresponding funnel plot for HbA1 can be seen in Figure S4A of the Supplementary Materials. Most studies are clustered near the top of the plot, indicating smaller standard errors, and are symmetrically distributed around the central effect size. However, two studies show extreme effect sizes, with one study far to the left (around −25.0) and another at approximately −5.0. The *p*-value obtained in the Egger test was *p* < 0.001. Regarding the risk of bias by families, (Figure S4B,C), the Zingiberaceae studies showed no significant evidence of publication bias according to Egger’s test (β = −3.74; β SE = 2.46; z-value = −1.52; *p* = 0.13), and the funnel plot showed a relatively balanced distribution of studies around the estimated effect size; in contrast, the Berberidaceae studies showed substantial small-study effects and a clear presence of bias, as indicated by both the funnel plot and Egger’s test results (β = −25.83; β SE = 5.66; z-value = −4.56; *p* < 0.001).

**Figure 7:**
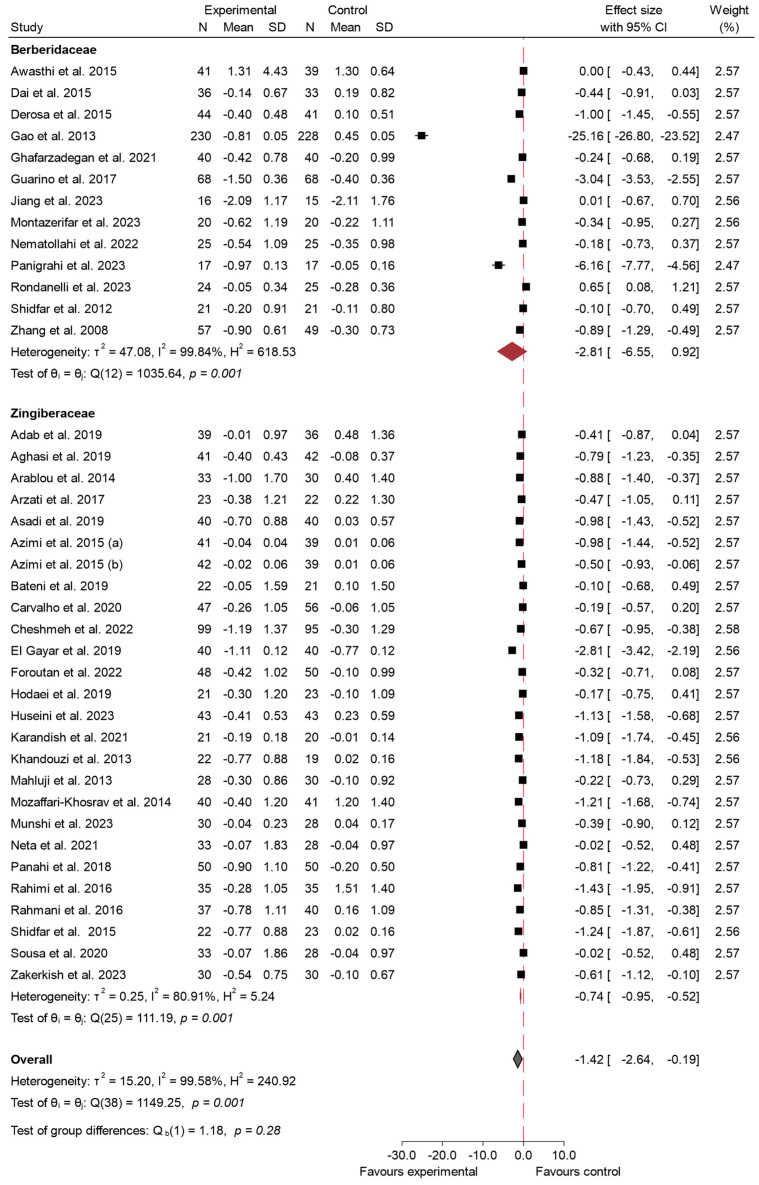
Subgroup analysis in relation to family group for HbA1c [5,16,17,19,20,21,22,23,25,28,29,31,34,35,37,38,39,42,43,45,46,48,50,52,53,54,55,56,58,59,61,63,65,68,69,71,73].

When analyzing the effects on HbA1c by family, the results demonstrated substantial differences between the Berberidaceae and Zingiberaceae species. For Berberidaceae, the pooled effect size was −2.81 (95% CI: −6.55 to 0.92; *p* < 0.001). This indicates that the effect of consuming species from the Berberidaceae family on HbA1c is not clinically relevant, as the confidence interval crosses the line of null effect, suggesting a negligible or non-existent impact in this case. Additionally, heterogeneity among the studies was high, with an *I*^2^ = 99.84%, indicating notable variability across the included studies. In contrast, the Zingiberaceae species showed a pooled effect size of −0.74 (95% CI: −0.95 to −0.52; *p* < 0.001), indicating a significant reduction in HbA1c levels. Heterogeneity for Zingiberaceae studies was lower (*I*^2^ = 80.91%), suggesting more consistency in the outcomes. The comparison between the two families, however, did not show a significant difference in effect sizes (*p* = 0.28), as indicated in Figure 7.

#### 3.4.3. Insulin

**Figure 8:**
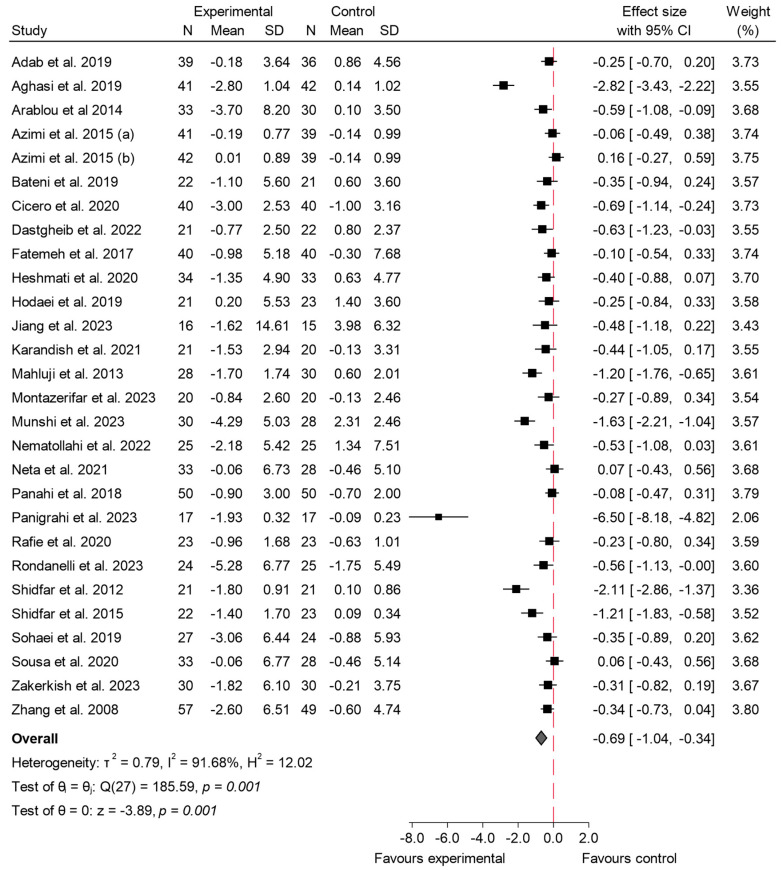
Comparisons of the effects of consumption of the Zingiberaceae and Berberidaceae species versus a control on insulin [5,16,17,19,23,25,30,33,36,41,42,45,46,50,52,54,55,56,58,59,60,65,68,69,70,71,73].

A total of 26 studies explored the impact of 31 long-term studies [5,16,17,19,23,25,29,30,33,35,36,41,42,45,46,50,52,54,55,56,58,59,60,64,65,66,68,69,70,71,73] on the consumption of species from the Zingiberaceae and Berberidaceae families on insulin levels. The overall meta-analysis revealed a significant reduction in this variable in patients who consumed any of the experimental products, with an effect size of −0.75 (95% CI: −1.13 to −0.38; *p* < 0.001; *I*^2^ of 91.87%). These results are shown in Figure 8. Meta-regression did not reveal a significant association between either the administered dose (β = −0.059; *p* = 0.564) or the intervention duration (β = −0.046; *p* = 0.380) and the effect size. These findings suggest that neither variable contributed meaningfully to the observed heterogeneity, and the model explained only a small proportion of the between-study variance.

The results were further assessed through a sensitivity analysis (Figure S5) by excluding outlier studies [23]. Following this exclusion, the overall effect size adjusted slightly to −0.80 (95% CI: −1.17 to −0.43; *p* < 0.002), indicating a stable reduction in insulin levels despite the removal of these studies. Despite this, heterogeneity remained high (*I*^2^ = 88.7%), demonstrating only a minor improvement in consistency among the studies. The funnel plot corresponding to insulin (Figure S6A) suggested a slight asymmetry, with smaller studies tending to show larger effects. Egger’s test (β = −9.46, SE = 1.37, z = −6.91, *p* < 0.001) also highlighted potential small-study effects or publication bias. This asymmetry remained evident for both the Berberidaceae and Zingiberaceae species (Figure S6B,C of the Supplementary Materials), suggesting that the effects of small studies are not limited to a particular family (Berberidaceae: *p* < 0.001; Zingiberaceae: *p* < 0.003).

Upon examining the data by family, notable differences in outcomes were observed. In the case of the Berberidaceae family, the effect size was −1.45 (95% CI: −2.98 to 0.09; *p* = 0.09). Additionally, the heterogeneity for the Berberidaceae studies was exceptionally high (*I*^2^ = 97.70%), indicating a wide variation in the study results.

On the other hand, the Zingiberaceae family demonstrated a more robust and consistent reduction in insulin levels. The pooled effect size was −0.52 (95% CI: −0.81 to −0.24; *p* < 0.001), showing a statistically significant decrease. This group also exhibited a comparatively lower degree of heterogeneity (*I*^2^ = 85.05%), suggesting more uniformity in the study outcomes for these species.

**Figure 9:**
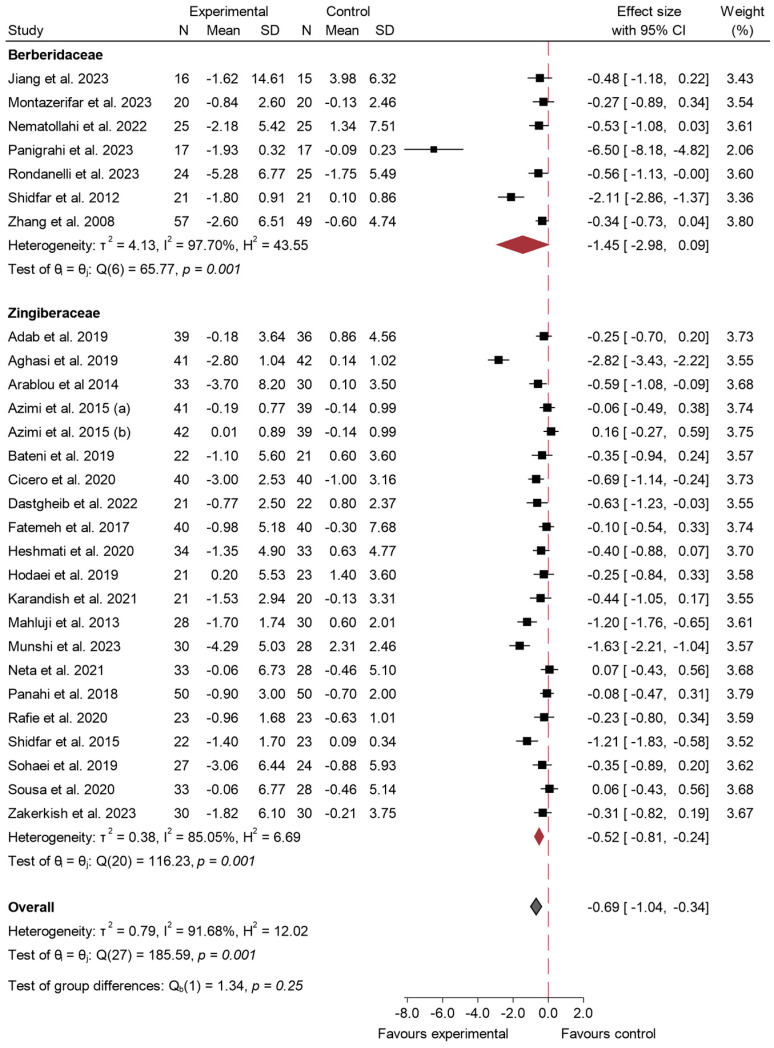
Subgroup analysis in relation to the family group for insulin [5,16,17,19,23,25,30,33,36,41,42,45,46,50,52,54,55,56,58,59,60,65,68,69,70,71,73].

When comparing the two families, the analysis did not reveal a statistically significant difference between their effect sizes (*p* = 0.25). Although variations in the magnitude of the effect were observed, these differences were not statistically meaningful (Figure 9).

#### 3.4.4. HOMA-IR

**Figure 10:**
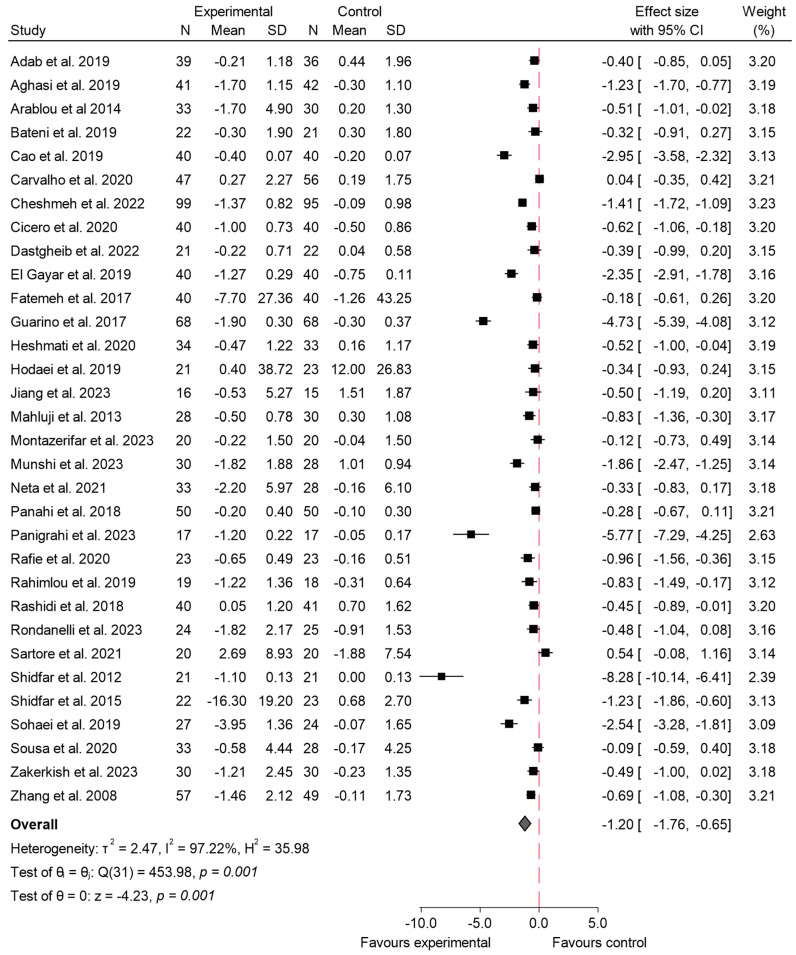
Comparisons of the effects of consumption of Zingiberaceae and Berberidaceae species versus a control on HOMA-IR index [5,16,17,19,25,27,28,29,30,33,35,40,41,42,45,50,52,54,56,58,59,60,62,64,65,68,69,70,71,73].

In Figure 10, the results of the meta-analysis for the variable HOMA-IR can be observed [5,16,17,19,25,27,28,29,30,33,35,40,41,42,45,50,52,54,56,58,59,60,62,64,65,68,69,70,71,73]. The overall effect size was −1.20 (95% CI: −1.76 to −0.65; *p* < 0.001; *I*^2^ = 97.22%), indicating a significant reduction in insulin resistance in the experimental group compared to the control. Despite this percentage of heterogeneity, the analysis consistently demonstrated that the consumption of species from the Zingiberaceae and Berberidaceae families led to a significant improvement in HOMA-IR values compared to the control group. The meta-regression analysis identified a significant inverse association between intervention duration and effect size (β = −0.081; *p* < 0.013), suggesting that longer interventions were linked to greater improvements in insulin resistance. Conversely, no significant association was observed for dose (β = −0.104; *p* = 0.492). This model accounted for 17.83% of the heterogeneity across studies.

The overall effect size remained significant at −1.14 (95% CI −1.71 to −0.58; *p* < 0.001) after sensitivity analysis excluding the one study identified as having a high risk of bias [27]. Heterogeneity remained high (*I*^2^ = 97.21%), indicating considerable variability between studies. This can be seen in Figure S7 in the Supplementary Materials. In addition, funnel plots were generated. The plots for the total sample studies (Figure S8A) and the Berberidaceae (Figure S8B) show a remarkable asymmetry, suggesting a possible publication bias. In contrast, the funnel plot of Zingiberaceae (Figure S8C) appears more symmetrical. Egger’s tests corroborate these results, since statistically significant differences were observed in all of them (Global: *p* < 0.001; Berberidaceae *p* < 0.001; Zingiberaceae *p* < 0.03).

**Figure 11:**
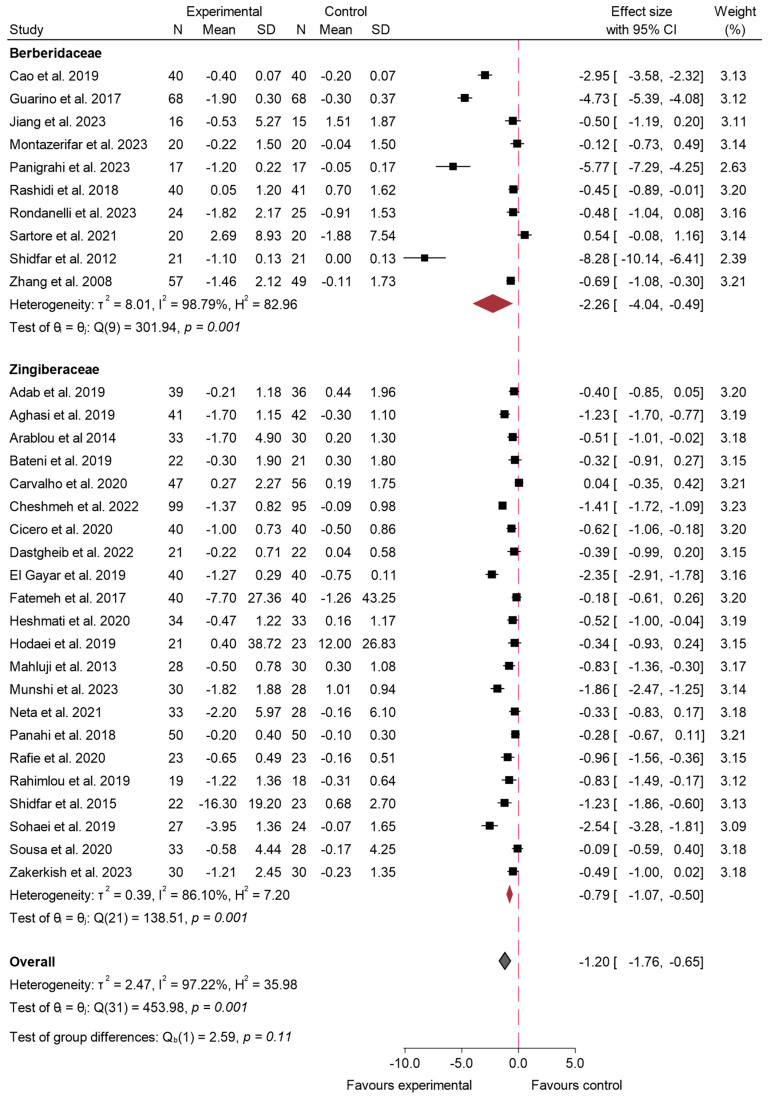
Subgroup analysis in relation to family group for HOMA-IR index [5,16,17,19,25,27,28,29,30,33,35,40,41,42,45,50,52,54,56,58,59,60,62,64,65,68,69,70,71,73].

The subgroup analysis, as shown in Figure 11, revealed different effects between the two plant families. For Berberidaceae, the pooled effect size was −2.26 (95% CI: −4.04 to −0.49; *p* < 0.001), with these results showing a null effect for the observed reduction. In contrast, the effect of Zingiberaceae was significant and consistent, with an effect size of −0.79 (95% CI: −1.07 to −0.50; *p* < 0.001), indicating a significant reduction in HOMA-IR levels. Although the effect seems larger for Zingiberaceae, the statistical comparison between the two families did not reach significance (*p* = 0.10). High heterogeneity was observed in both subgroups, with *I*^2^ = 98.79% for Berberidaceae and *I*^2^ = 86.10% for Zingiberaceae.

#### 3.4.5. 2hPG

**Figure 12:**
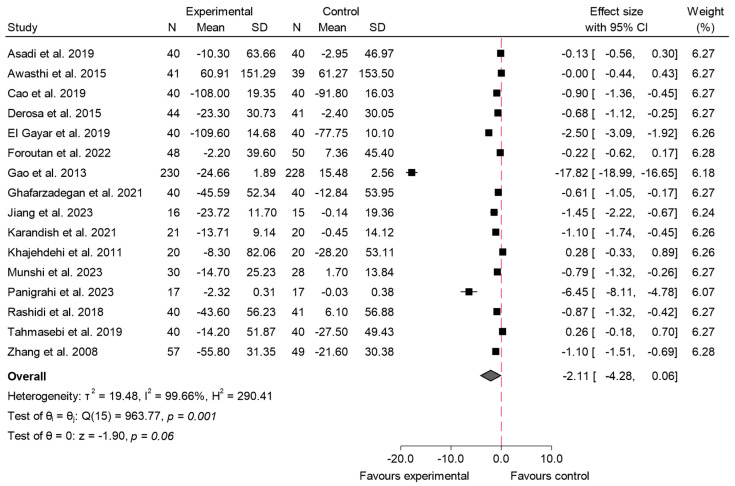
Comparisons of the effects of consumption of the Zingiberaceae and Berberidaceae species versus a control on 2hPG [5,21,22,27,34,35,37,38,39,45,46,47,54,59,64,72].

In Figure 12, the results of the meta-analysis for the variable 2hPG are presented [5,21,22,27,34,35,37,38,39,45,46,47,54,59,64,72]. The overall effect size was −2.11 (95% CI: −4.28 to 0.06; *p* = 0.06; *I*^2^ = 99.66%), indicating that the consumption of species from the Zingiberaceae and Berberidaceae families did not produce a statistically significant effect on postprandial glucose levels in the studies analyzed. For this variable, meta-regression analysis showed that both dose and duration significantly contributed to the magnitude of the effect. Specifically, greater doses (β = −0.697; *p* < 0.016) and longer interventions (β = −0.215; *p* < 0.044) were associated with more pronounced reductions in 2hPG. Notably, this model accounted for 52.7% of the heterogeneity, indicating a substantial proportion of the variability was explained by these factors.

Sensitivity analysis for the variable 2hPG, shown in the forest plot in Figure S9 (excluding three studies [22,27,37]), reveals a combined effect size of −2.51 (95% CI: −5.16 to 0.13; *I*^2^ = 99.68%). Despite the high heterogeneity, the overall trend suggests a beneficial effect of the experimental intervention compared to the control, although it is not statistically significant (*p* = 0.06).

The publication bias can be seen in Figure S10. It shows great visual heterogeneity both in the overall sample and in the studies on Berberidaceae. In the studies on the consumption of species of the Zingiberaceae family, the plot is more symmetrical. Egger’s test was significant for the overall sample and the Berberidaceae (*p* < 0.001), while for the Zingiberaceae, a *p* value of 0.43 was obtained.

**Figure 13:**
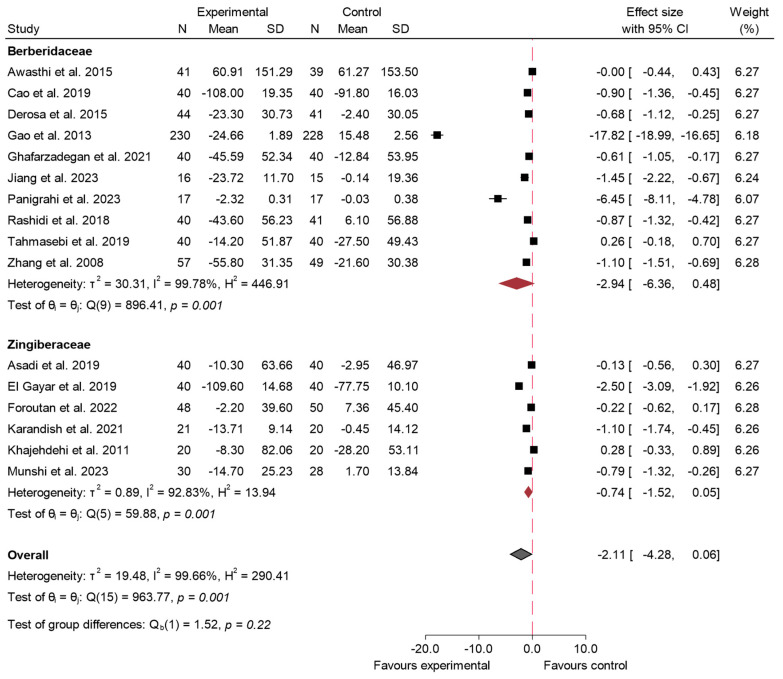
Subgroup analysis in relation to the family group for 2hPG [5,21,22,27,34,35,37,38,39,45,46,47,54,59,64,72].

Subgroup analysis for 2hPG is shown in Figure 13. This meta-analysis showed no statistically significant effect for any of the families on postprandial glucose levels for this family (*p* = 0.22) (Berberidaceae: effect size −2.94; 95% CI: −6.36 to 0.48; *I*^2^ = 99.78%; Zingiberaceae: effect size −0.74; 95% CI: −1.52 to 0.05; *I*^2^ = 92.83%).

#### 3.4.6. Body Weight and BMI

**Figure 14:**
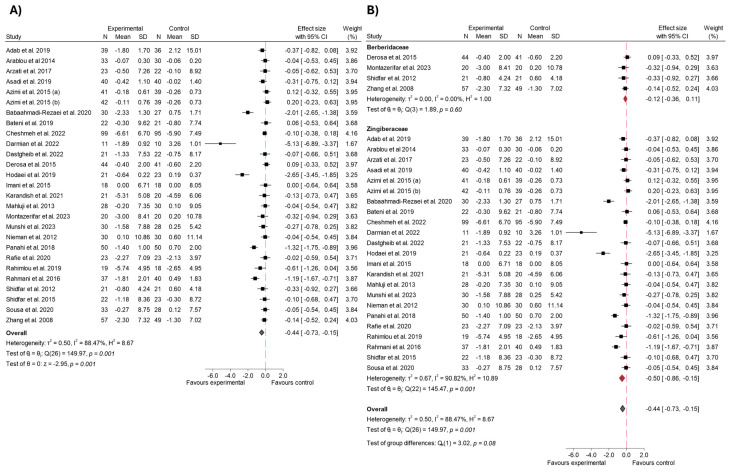
(**A**) Comparisons of the effects of consumption of the Zingiberaceae and Berberidaceae species versus a control on body weight; (**B**) subgroup analysis in relation to family group for body weight [5,16,19,20,21,23,24,25,29,32,33,34,41,42,44,46,50,52,54,57,58,60,62,63,68,69,71].

**Figure 15:**
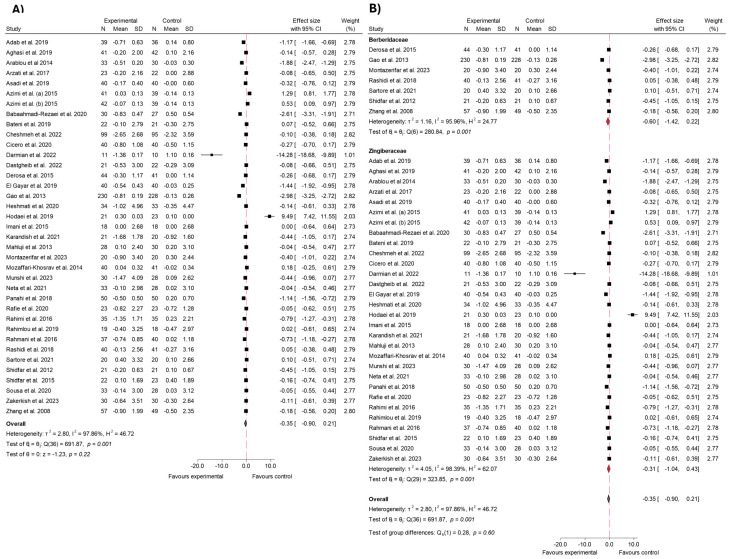
(**A**) Comparisons of the effects of consumption of the Zingiberaceae and Berberidaceae species versus a control on BMI; (**B**) subgroup analysis in relation to the family group for BMI [5,16,17,19,20,21,23,24,25,29,30,32,33,34,35,38,41,42,44,46,50,52,53,54,56,58,60,61,62,63,64,66,68,69,71,73].

The results of the meta-analysis for body weight are shown in Figure 14A. The overall effect size was −0.44 (95% CI: −0.73 to −015; *p* < 0.001), indicating a statistically significant reduction in body weight in the experimental group compared to the control group [5,16,19,20,21,23,24,25,29,32,33,34,41,42,44,46,50,52,54,57,58,60,62,63,68,69,71]. Moderate heterogeneity was observed among the studies (*I*^2^ = 88.47%), although the general trend points to a consistent reduction in body weight. In contrast, for the BMI variable, no statistically significant differences were observed for the studies analyzed since the estimated effect size was −0.35 (95% CI: −0.90 to 0.21; *p* = 0.22; *I*^2^ = 97.86%) [5,16,17,19,20,21,23,24,25,29,30,32,33,34,35,38,41,42,44,46,50,52,53,54,56,58,60,61,62,63,64,66,68,69,71,73]. This can be seen in Figure 15A.

Regarding anthropometric outcomes, meta-regression analyses for both body weight and BMI did not reveal statistically significant associations with either intervention duration or dosage (*p* > 0.05). These findings suggest that, in the included studies, variations in dose or length of the intervention were not consistent predictors of changes in weight or BMI. This may reflect the influence of other unmeasured factors or the limited sensitivity of these outcomes to the interventions assessed.

Subgroup analysis for body weight, as shown in Figure 14B, highlights differences between families. For Berberidaceae, the pooled effect size was −0.12 (95% CI: −0.36 to 0.11; *p* = 0.60). Furthermore, heterogeneity for this subgroup was low (*I*^2^ = 0.0%), suggesting consistency in the results across studies. In contrast, the Zingiberaceae family showed a significant reduction in body weight, with an effect size of −0.50 (95% CI: −0.86 to −0.15; *p* < 0.001). However, there was considerable heterogeneity in this subgroup (*I*^2^ = 90.82%), reflecting variability in individual study results. Despite the apparent differences between the two subgroups, the test for differences between subgroups was not statistically significant (*p* = 0.08), indicating that the effect of species from both families on body weight may not differ substantially.

The subgroup analysis for BMI (Figure 15B) revealed no statistically significant differences between the two families (*p* = 0.60). For the Berberidaceae family, the effect size was −0.60 (95% CI: −1.42 to 0.22; *I*^2^ = 95.96%), while for the Zingiberaceae family, the effect size was −0.31 (95% CI: −1.04 to 0.43; *I*^2^ = 98.39%).

In the sensitivity analysis for the weight variable, no substantial changes in effect size were observed after excluding studies with a high risk of bias (effect size: −0.40; 95% CI: −0.65 to −0.14; *I*^2^ = 83.25%) [23,32]. For the BMI variable, the effect size remained nearly unchanged (effect size: −0.30; 95% CI: −0.75 to 0.15; *I*^2^ = 96.58%; study excluded: [23,32]). These findings are illustrated in Figure S11.

Figure S12 shows funnel plots for body weight: (A) all studies and subgroup analyses for (B) Berberidaceae and (C) Zingiberaceae species. The overall plot is relatively symmetrical, indicating a low risk of publication bias. Subgroup plots (B and C) show some asymmetry, likely due to the smaller number of studies, which limits bias assessment. Figure S13 shows the funnel plots for BMI. Similarly to body weight, the overall plot (A) shows a relatively symmetrical distribution, suggesting a low risk of publication bias. In the subgroup plots, (B) shows some asymmetry, potentially due to the limited number of studies in this category, while (C) appears relatively symmetrical.

## 4. Discussion

This systematic review and meta-analysis included data from 58 studies that evaluated the effects of species from the Zingiberaceae and Berberidaceae families on various glycemic parameters, including FBS, HbA1c, fasting insulin levels, and HOMA-IR. Our findings demonstrate significant reductions in these parameters, suggesting that these bioactive compounds may serve as valuable adjuncts in managing glycemic control, particularly in individuals with altered glucose metabolism, such as those with T2 DM or insulin resistance.

As previously mentioned, curcumin and structurally related diarylheptanoids from the Zingiberaceae family have demonstrated significant therapeutic potential in glycemic control due to their antidiabetic, anti-inflammatory, and antioxidant properties [74]. Their biological activity is largely attributed to their ability to modulate multiple molecular targets and pathways. For instance, curcumin has been shown to activate AMPK, thereby enhancing glucose uptake and fatty acid oxidation in peripheral tissues while suppressing hepatic gluconeogenesis [75]. Additionally, it inhibits nuclear factor-kappa B (NF-κB) signaling, leading to reduced expression of pro-inflammatory cytokines such as TNF-α and IL-6, which are implicated in insulin resistance [76]. Structurally, diarylheptanoids feature a distinctive 1,7-diphenylheptane backbone that allows for substantial chemical diversity through modifications in methoxy and hydroxyl substitutions, as well as conjugation with other moieties [77]. This structural variability underpins their broad pharmacological profile, including the radical scavenging capacity, modulation of adipokines, and preservation of pancreatic β-cell function [78]. Recent advances in synthetic chemistry have focused on improving the bioavailability and stability of curcumin analogs. For example, synthetic derivatives such as EF24 have demonstrated enhanced solubility and biological potency in preclinical models of diabetes and metabolic syndrome [79]. These efforts are in line with recent comprehensive reviews highlighting key methodologies for diarylheptanoid synthesis, structural modifications, and pharmacological profiling over the past decade [80,81].

Likewise, berberine and other isoquinoline alkaloids, representative of the Berberidaceae family, have demonstrated consistent glucose-lowering effects in randomized controlled trials, with proposed mechanisms including AMPK activation, inhibition of mitochondrial respiratory complex I, suppression of hepatic gluconeogenesis, and enhancement of GLUT4 translocation [82,83]. In addition, berberine modulates gut microbiota composition and bile acid metabolism, further contributing to improved insulin sensitivity and glucose homeostasis [84]. Phytochemically, berberine and related alkaloids such as jatrorrhizine, palmatine, and coptisine share a tetracyclic protoberberine skeleton, with a quaternary nitrogen that facilitates intercalation into nucleic acids, inhibition of protein kinases, and modulation of transcription factors like PPAR-γ [85]. These structural characteristics enhance their multi-targeted pharmacological effects, not only on glycemic parameters but also on inflammation and oxidative stress.

Taken together, the compounds discussed above exhibit a rich phytochemical diversity with the capacity to act through convergent and complementary pathways. This molecular complementarity may explain the consistent effects observed across different studies and justify the botanical family-based approach adopted in this meta-analysis. Beyond phytochemical mechanisms, several additional factors, including methodological heterogeneity, population characteristics, and intervention quality, must be considered to fully contextualize the findings and assess their clinical applicability. These aspects are discussed in the following sections.

### 4.1. Effects on Fasting Blood Glucose (FBS)

Our meta-analysis revealed that the consumption of species from both the Zingiberaceae and Berberidaceae families significantly reduced FBS levels, with an overall effect size of −1.06 (95% CI: −1.42 to −0.71; *p* < 0.001). This aligns with the established hypoglycemic effects of compounds like berberine [86] and curcumin [87], which are known to improve insulin sensitivity and inhibit hepatic gluconeogenesis [88,89]. Berberine has been shown to reduce glucose production in the liver and increase glucose uptake in peripheral tissues by activating AMP-activated protein kinase (AMPK) [90], which plays a critical role in regulating cellular energy homeostasis. Additionally, curcumin’s anti-inflammatory and antioxidant properties contribute to its potential to lower blood glucose by reducing oxidative stress and improving pancreatic β-cell function [91], which is often impaired in individuals with T2 DM.

Subgroup analysis showed that the Berberidaceae species, particularly those containing berberine, had a more pronounced effect on FBS reduction (effect size: −1.90; 95% CI: −2.77 to −1.04), compared to the Zingiberaceae species (effect size: −0.65; 95% CI: −0.91 to −0.39). This difference could be attributed to the more potent mechanisms of action of berberine, which include the activation of AMPK, enhancement of glucose transporter-4 (GLUT-4) activity in muscle and adipose tissue, and inhibition of hepatic gluconeogenesis through the downregulation of key enzymes such as phosphoenolpyruvate carboxykinase (PEPCK) and glucose-6-phosphatase [92]. Additionally, berberine has been shown to modulate gut microbiota [93], which may further enhance glucose metabolism by increasing the production of short-chain fatty acids (SCFAs), thereby improving insulin sensitivity and reducing systemic inflammation.

On the other hand, the Zingiberaceae species, including ginger and turmeric, exhibited a significant but less pronounced reduction in FBS levels. These species exert their effects through several pathways, including the inhibition of inflammatory pathways like nuclear factor-kappa B (NF-κB) and the modulation of insulin sensitivity via the enhancement of insulin receptor signaling [94]. Curcumin, the active compound in turmeric, has also been demonstrated to suppress the inflammatory cytokines that contribute to insulin resistance [95], which could explain its beneficial effects on glucose metabolism, though to a lesser degree than berberine. In the case of ginger, bioactive compounds such as gingerol and shogaol have been shown to inhibit key enzymes like α-glucosidase and α-amylase, which are involved in carbohydrate metabolism, thus potentially reducing serum glucose levels [96]. Additionally, gingerols enhance glucose uptake in skeletal muscle cells by promoting the translocation of the glucose transporter GLUT4 to the plasma membrane [97]. Despite these mechanisms, the results from various studies have shown mixed effects on FBS reduction. For instance, trials by Arzati et al. [20] reported significant decreases in FBS and HbA1c with daily supplementation of 2 g of ginger over 10–12 weeks, while others, like Mahluji et al. [50], found no substantial changes in FBS levels (*p* = 0.42) with shorter intervention durations and varying dosages. These discrepancies may be related to differences in the composition of ginger extracts, preparation methods, and baseline characteristics of the participants.

These results are consistent with previous meta-analyses that investigated isolated compounds. For instance, Xie et al. [83] reported a significant reduction in fasting plasma glucose of −0.82 mmol/L (95% CI: −0.95 to −0.70) in patients treated with berberine. Regarding curcumin, Mokgalaboni et al. [98] found a decrease of −11.48 mg/dL (approximately −0.64 mmol/L).

However, significant heterogeneity (*I*^2^ = 98.45%) was observed across studies, likely due to variations in dosages, intervention durations, and participant characteristics. For instance, studies on Berberidaceae often used higher doses of berberine (up to 3 g/day), whereas the Zingiberaceae species were administered in relatively lower doses (ranging from 1 to 2 g/day for ginger and turmeric). Furthermore, the duration of the interventions varied considerably, with some studies lasting only 4 weeks, while others extended beyond 12 weeks, potentially influencing the degree of FBS reduction observed. The baseline characteristics of participants, such as the severity of their glycemic dysregulation, BMI, and concurrent use of antidiabetic medications, may also have contributed to the observed heterogeneity.

### 4.2. Impact on HbA1c

HbA1c, a marker of long-term glycemic control, was significantly reduced in the studies included in our analysis, with an overall effect size of −1.42 (95% CI: −2.64 to −0.19; *p* < 0.02). This finding underscores the importance of these bioactive compounds in the management of T2 DM over extended periods, as HbA1c reflects average blood glucose levels over the preceding 2–3 months. The observed reduction in HbA1c exceeds the threshold commonly considered clinically meaningful. According to the American Diabetes Association, a decrease of at least 0.5% is associated with improved glycemic control and reduced risk of long-term complications in individuals with type 2 diabetes mellitus [99]. As mentioned previously, the more pronounced reductions seen with the Berberidaceae species, such as berberine, could be attributed to its stronger mechanisms of action, including the activation of AMPK and improvement in insulin receptor expression, which have been shown to directly impact long-term glycemic control [92]. Similarly, Liang et al. [100] conducted a comprehensive meta-analysis involving 28 studies and 2313 participants, confirming that berberine effectively reduced fasting plasma glucose (WMD = −0.54 mmol/L, 95% CI: −0.77 to −0.30) and postprandial plasma glucose (WMD = −0.94 mmol/L, 95% CI: −1.27 to −0.61) levels, while also lowering HbA1c levels. Consistent with previous findings, Liang et al. attributed these benefits to berberine’s activation of AMPK.

The effect size observed with the Berberidaceae species (effect size: −2.81; 95% CI: −6.55 to 0.92) suggests variability between studies. The wide confidence interval, which includes the null effect, indicates that the reduction in HbA1c associated with these species is not statistically significant. This variability could be linked to differences in intervention length, dosage, and participant characteristics across the included trials. Berberine, as highlighted in prior research, is known to effectively reduce HbA1c by modulating glucose metabolism and improving insulin sensitivity, mechanisms that are likely to explain these results [86]. Nevertheless, the wide confidence interval signals that more uniform methodologies and controlled dosages would help in confirming these findings more conclusively.

On the other hand, the impact of the Zingiberaceae species, including turmeric and ginger, showed a statistically significant effect size of −0.74 (95% CI: −0.95 to −0.52). The confidence interval does not include the null effect, indicating a consistent reduction in HbA1c. As noted previously, compounds such as curcumin and gingerols exert hypoglycemic effects by enhancing insulin sensitivity and reducing inflammation. Despite the smaller magnitude of the effect compared to Berberidaceae, the narrower confidence interval reflects lower variability and greater reliability among the studies involving Zingiberaceae species [88,90].

The magnitude of HbA1c reduction observed in this review aligns with earlier data from targeted interventions. According to Xie et al. [83], berberine reduced HbA1c by −0.63% (95% CI: −0.72 to −0.53) in individuals with type 2 diabetes. Similarly, curcumin supplementation led to a reduction of −0.54% (95% CI: −0.73 to −0.35) in a meta-analysis by Mokgalaboni et al. [98].

The considerable heterogeneity observed in this meta-analysis (*I*^2^ = 99.58%) likely stems from the varying study designs, participant characteristics, and intervention strategies. As seen in other glycemic measures, baseline HbA1c levels can significantly influence the degree of reduction observed, with higher baseline levels generally leading to more pronounced decreases in response to the intervention. In addition, the duration of the studies and the form in which the supplements were administered (e.g., standardized extracts, nano-formulations, or crude plant powders) could have contributed to the variability in the findings.

As previously discussed with FBS, the bioavailability of active compounds, particularly in the Zingiberaceae species like curcumin, may also play a critical role in the observed outcomes. Enhanced formulations, such as those including piperine to increase curcumin absorption [101], were used in some studies, which may have led to more favorable outcomes, whereas studies using less bioavailable forms might show diminished effects. This variability points to the potential of optimized formulations in improving glycemic control outcomes and underscores the need for further trials that assess these more advanced preparations.

### 4.3. Impact on Insulin Levels

The studies included in this meta-analysis demonstrated a significant reduction in fasting insulin levels among participants who consumed species from the Zingiberaceae and Berberidaceae families, with an overall effect size of −0.75 (95% CI: −1.13 to −0.38; *p* < 0.001). This decrease in insulin levels indicates an improvement in insulin sensitivity, which is crucial for managing T2 DM and insulin resistance.

Similarly, a comprehensive meta-analysis by Zamani et al. [102] found that berberine supplementation significantly lowered fasting insulin levels (WMD = −3.27 mg/dL; 95% CI: −4.46 to −2.07; *p* < 0.001), demonstrating its effectiveness in improving insulin resistance. This reduction is primarily attributed to berberine’s ability to activate AMPK, which enhances glucose uptake, reduces hepatic glucose production, and increases insulin receptor expression.

On the other hand, the Zingiberaceae species demonstrated a smaller yet significant effect (−0.52; 95% CI: −0.81 to −0.24), consistent with previous research. A systematic review and meta-analysis by Zhu et al. [10] confirmed that ginger supplementation significantly reduced fasting insulin levels, with a pooled effect size of −1.62 µIU/mL (95% CI: −2.20 to −1.05; *p* < 0.001). This reduction suggests that ginger improves insulin sensitivity, making it an effective adjuvant therapy for individuals with type 2 diabetes and metabolic syndrome. This study highlighted that ginger’s active components, such as 6-gingerol and 6-shogaol, play key roles in enhancing glucose metabolism and insulin signaling, leading to better glycemic control and reduced insulin resistance. Despite this, the moderate reduction observed with the Zingiberaceae species suggests that their bioactive compounds might be less potent in targeting insulin resistance compared to berberine.

However, the significant heterogeneity observed in this analysis (*I*^2^ = 91.87%) is likely due to variations in dosages, intervention durations, and participant characteristics.

### 4.4. Impact on HOMA-IR

HOMA-IR, a key marker of insulin resistance, showed significant reduction across the studies analyzed, with an overall effect size of −1.20 (95% CI: −1.76 to −0.65; *p* < 0.001). This reduction is critical, as insulin resistance plays a central role in the pathogenesis of T2 DM. The effect was particularly notable in studies involving the Berberidaceae species, with a pooled effect size of −2.26 (95% CI: −4.04 to −0.49), supporting the hypothesis that berberine exerts more potent effects on insulin sensitivity through multiple pathways, including the activation of AMPK and modulation of gut microbiota.

A meta-analysis by Ye et al. [6] also supported these findings, demonstrating that berberine supplementation significantly improved HOMA-IR in individuals with metabolic syndrome and T2 DM, with a standardized mean difference (SMD) of −1.25 (95% CI: −2.24 to −0.25; *p* = 0.01).

Conversely, the effect of the Zingiberaceae species, while significant (−0.79; 95% CI: −1.07 to −0.50), was relatively smaller. Supporting this, a randomized controlled trial by Chuengsamarn et al. [103] demonstrated that curcumin significantly reduced HOMA-IR in a prediabetic population over a 9-month intervention period. The study attributed these benefits to curcumin’s ability to increase adiponectin levels and reduce pro-inflammatory cytokines, thereby enhancing insulin sensitivity. Additionally, the meta-analysis conducted by Melo et al. found a mean decrease of −1.26 (95% CI: −3.71 to 1.19) with curcumin, although not statistically significant (*p* = 0.31) [104]. Nevertheless, the high heterogeneity observed in this meta-analysis (*I*^2^ = 97.22%) indicates variability in study designs, durations, and baseline characteristics, which could significantly influence these outcomes.

### 4.5. Impact on 2hPG

The meta-analysis of studies evaluating 2hPG did not show a statistically significant effect from the consumption of species from either the Zingiberaceae or Berberidaceae families, with an overall effect size of −2.11 (95% CI: −4.28 to 0.06; *p* = 0.06). These mixed results were attributed to differences in dosages, formulations, and baseline glucose levels, which can heavily influence the postprandial response.

However, several individual studies on ginger supplementation have reported more favorable outcomes. For example, Mozaffari-Khosravi et al. [53] found significant reductions in postprandial glucose among T2 DM patients after 12 weeks of ginger supplementation, likely due to ginger’s inhibition of key digestive enzymes such as α-amylase and α-glucosidase. Similarly, studies on berberine have shown improvements in 2hPG, potentially through its effects on gut microbiota and insulin-mediated glucose uptake [83]. For example, Xie et al. [83] found a significant reduction of −1.16 mmol/L (95% CI: −1.36 to −0.96) with berberine supplementation.

The lack of consistent results in this meta-analysis suggests the need for standardized study designs and intervention protocols to clarify the true impact of these bioactive compounds on postprandial glucose levels.

### 4.6. Impact on Body Weight and BMI

Obesity is a key factor in the development of numerous metabolic and non-metabolic disorders, including coronary heart disease, T2 DM, and various types of cancer [105,106]. Our meta-analysis findings showed that the consumption of Zingiberaceae and Berberidaceae species led to a significant reduction in body weight, with an overall effect size of −0.44 (95% CI: −0.73 to −0.15; *p* < 0.001). This decrease in weight may be linked to the improvement in insulin sensitivity and a reduction in inflammatory markers, as well as the potential thermogenic properties of specific bioactive compounds present in these species [92,107]. However, the impact on BMI was not statistically significant in this meta-analysis, with high heterogeneity (*I*^2^ = 97.86%). These results could be explained by variations in muscle mass, water content, and other factors that can influence BMI independently of fat loss.

Supporting these findings, a meta-analysis by Maharlouei et al. [108] demonstrated inconsistent effects of ginger on BMI across studies, suggesting that changes in BMI alone might not accurately reflect alterations in body composition. Therefore, incorporating additional parameters like waist-to-hip ratio (WHR) or body fat percentage could provide a more comprehensive assessment. Similarly, Asbaghi et al. [109] conducted a meta-analysis focusing on berberine supplementation, which showed moderate but significant reductions in weight, BMI, and waist circumference, along with lowered markers of inflammation.

Comparatively, the Zingiberaceae species exhibited more consistent reductions in body weight, while the Berberidaceae species, primarily berberine, demonstrated mixed results on BMI. The weight-loss potential of the Zingiberaceae species, particularly ginger, has been corroborated by several studies. For instance, supplementing with 2 g/day of ginger over 12 weeks resulted in a notable decline in BMI among obese women [110]. On the other hand, Unhapipatpong et al. noted modest weight changes with curcumin in individuals with metabolic conditions, including T2 DM [111]. Moreover, compounds like curcumin [112] in turmeric or berberine in the Berberidaceae species [113] have been shown to promote thermogenesis and fat breakdown, which can further aid in weight loss and fat reduction. These mechanisms, combined with enhanced insulin sensitivity, highlight the potential of these species for managing weight in individuals with metabolic conditions. Nonetheless, the high heterogeneity observed across studies underscores the need for standardized intervention protocols and more precise measures of body composition in future research to provide clearer insights into the efficacy of these interventions.

### 4.7. Mechanistic Summary of Glycemic Effects

**Figure 16:**
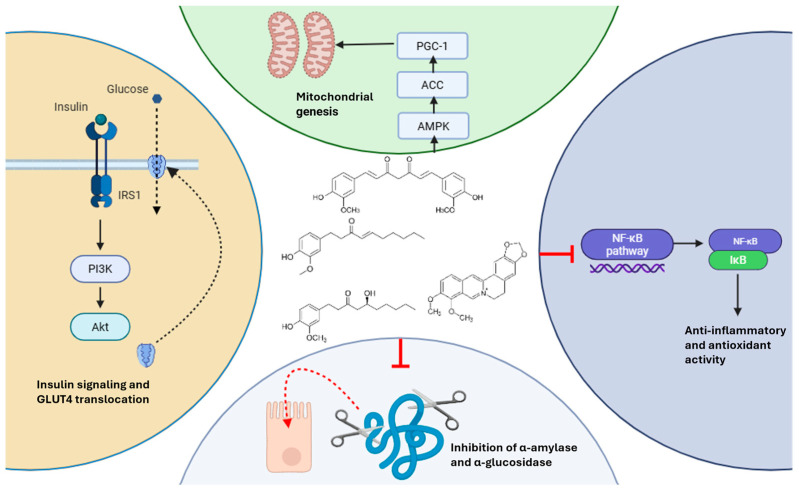
Proposed mechanisms of action of major bioactive compounds from Zingiberaceae (curcumin, gingerol, shogaol) and Berberidaceae (berberine) in glycemic regulation. These compounds activate AMPK, leading to downstream phosphorylation of ACC and stimulation of PGC-1α, thereby promoting mitochondrial biogenesis and improving cellular energy metabolism. They also enhance insulin signaling by stimulating the IRS-1/PI3K/Akt pathway, which promotes GLUT4 translocation and increases glucose uptake in muscle and adipose tissue. Additionally, curcumin, berberine, and gingerols exert inhibitory effects on α-amylase and α-glucosidase, delaying carbohydrate absorption and reducing postprandial glycemic spikes. Their anti-inflammatory and antioxidant properties, mediated by inhibition of NF-κB and activation of Nrf2, further contribute to improved insulin sensitivity and metabolic control.

A schematic representation of the phytochemical structures and proposed mechanisms of action of representative compounds from the Zingiberaceae and Berberidaceae families is provided in Figure 16. This visual synthesis integrates their effects on pathways such as insulin signaling, AMPK activation, and enzyme inhibition (e.g., α-amylase and α-glucosidase), offering a mechanistic perspective to support the metabolic outcomes observed in this review.

### 4.8. Strengths and Limitations

This review presents a number of strengths. It includes a substantial number of randomized controlled trials and was conducted in accordance with PRISMA guidelines. The eligibility criteria were clearly defined, and subgroup analyses by plant family and glycemic outcome were performed to explore differences in efficacy. A standardized risk of bias assessment was applied using the RoB 2.0 tool, contributing to the overall methodological consistency and transparency of the review process.

One factor that may contribute to the variability observed across studies is the extraction method and solvent used, as these can significantly influence the phytochemical composition and biological activity of the plant extracts. For instance, ethanol tends to extract more non-polar compounds such as curcuminoids, while water favors polar constituents like flavonoid glycosides. Differences in time, temperature, and mechanical techniques (e.g., maceration, reflux, ultrasound-assisted extraction) may further affect the concentration of active compounds. These methodological inconsistencies may help explain differences in clinical outcomes and highlight the need for greater standardization in future research.

Nonetheless, several limitations should be considered. The inclusion of studies was restricted to English-language publications, which may have introduced language bias and limited the comprehensiveness of the evidence base. Considerable heterogeneity was observed across most outcomes, likely reflecting differences in study design, intervention protocols, and population characteristics. Although subgroup and sensitivity analyses were conducted, some variability remained. Additionally, most studies were rated as having “some concerns” in the risk of bias assessment, particularly in domains related to randomization and outcome reporting. Signs of potential publication bias were also detected, especially in the Berberidaceae subgroup. Finally, safety and tolerability data were not consistently reported, limiting conclusions regarding long-term use.

## 5. Conclusions

This meta-analysis revealed differential effects of the Berberidaceae and Zingiberaceae species on various metabolic parameters. Both families showed significant reductions in FBS, with less heterogeneity observed between studies in the Zingiberaceae species. For HbA1c, while the overall effect was statistically significant, subgroup analysis by family indicated that only Zingiberaceae reached significance. For insulin levels, only Zingiberaceae and the overall analysis showed statistically significant reductions. Improvements in HOMA-IR were significant in both the overall and subgroup analyses for both families. On the other hand, no significant effects were observed for 2hPG in either group. For weight, the overall analysis and Zingiberaceae demonstrated significant reductions, but no significant changes were observed for BMI in either group.

These results highlight the consistent efficacy of Zingiberaceae species on multiple outcomes. In contrast, although the effects of Berberidaceae were not statistically significant on some variables, the observed trends suggest that their consumption may be promising, with some studies reporting beneficial changes. However, further studies with more consistent intervention periods and doses are needed to confirm these potential benefits more conclusively.

## Figures and Tables

**Table 1:** PICOS criteria used for this meta-analysis.

Element	Description
P (Participants)	Individuals with alterations in glycemic profile
I (Intervention)	Consumption of species from the Zingiberaceae or Berberidaceae families or their extracted bioactives
C (Comparisons)	Control group receiving a placebo
O (Outcomes)	Changes in fasting plasma glucose, glycated hemoglobin (HbA1c), fasting insulin levels, HOMA-IR index, or any other parameter related to the glycemic profile
S (Study Design)	Randomized controlled trials

**Table 2:** Study characteristics.

Reference	Year	Country	Design RCT	N	Women, n (%)	Age (Years)	BMI (kg/m^2^)	Family; Extract	Daily Dosage	Duration	Participants’ Health Condition	Outcome
Adab et al. [16]	2019	Iran	Double blind, parallel.	75	39 (52.0%)	55.2 ± 7.4	29.4 ± 4.4	Zingiberaceae; Turmeric	2.1 g	8 weeks	T2 DM	HbA1c; FBS; Insulin; HOMA-IR; Body weight; BMI
Aghasi et al. [17]	2019	Iran	Double blind, parallel.	83	39 (47.0%)	53.6 ± 6.2	29.1 ± 3.2	Zingiberaceae; Green cardamom	3 g	10 weeks	T2 DM	HbA1c; FBS; Insulin; HOMA-IR; BMI
Amin et al. [18]	2015	Pakistan	Double blind, parallel.	126	0%	42.0 ± 13.2	27.8 ± 4.6	Zingiberaceae; Turmeric	2.4 g	8 weeks	Prediabetes; Dyslipidaemia or pre-hypertension	FBS
Arablou et al. [19]	2014	Iran	Double blind, parallel.	63	48 (76.2%)	52.3 ± 8.6	26.9 ± 3.5	Zingiberaceae; Ginger	1.6 g	12 weeks	T2 DM	HbA1c; FBS; Insulin; HOMA-IR; Body weight; BMI
Arzati et al. [20]	2017	Iran	Double blind, parallel.	45	34 (68.0%)	50.7 ± 8.5	29.6 ± 4.1	Zingiberaceae; Ginger	2 g	10 weeks	T2 DM	HbA1c; FBS; Body weight; BMI
Asadi et al. [21]	2019	Iran	Double blind, parallel.	80	70 (87.5%)	54.1 ± 6.3	31.1 ± 4.0	Zingiberaceae; NanoCurcumin	80 mg	8 weeks	T2 DM	HbA1c; FBS; Body weight; BMI; 2hPG
Awasthi et al. [22]	2015	India	Parallel, absence of blinding.	80	33 (41.3%)	45.6 ± 8.4	25.6 ± 2.9	Berberidaceae; B. aristata	500 mg	24 weeks	T2 DM	HbA1c; FBS; 2hPG
Azimi et al. (a) [23]	2015	Iran	Single blind, parallel.	80	50 (62.5%)	54.4 ± 1.4	28.7 ± 0.4	Zingiberaceae; Ginger	3 g	8 weeks	T2 DM	HbA1c; FBS; Insulin; Body weight; BMI
Azimi et al. (b) [23]	2015	Iran	Single blind, parallel.	80	49 (60.5%)	52.6 ± 1.7	28.7 ± 0.3	Zingiberaceae; Cardamom	3 g	8 weeks	T2 DM	HbA1c; FBS; Insulin; Body weight; BMI
Babaahmadi-Rezaei et al. [24]	2020	Iran	Double blind, parallel.	57	0	56.4 ± 7.7	27.0 ± 1.2	Zingiberaceae; Ginger	1.6 g	8 weeks	Atherosclerosis	FBS; Body weight; BMI
Bateni et al. [25]	2019	Iran	Double blind, parallel.	43	33 (76.7%)	52.0 ± 8.2	29.7 ± 4.4	Zingiberaceae; Curcumin	80 mg	12 weeks	Metabolic syndrome	HbA1c; FBS; Insulin; HOMA-IR; Body weight; BMI
Bupparenoo et al. [26]	2020	Thailand	Double blind, parallel.	39	15 (38.5%)	55.4 ± 10.9	27.1 ± 4.2	Zingiberaceae; Curcumin	1 g	8 weeks	Asymptomatic Hyperuricemia	FBS
Cao et al. [27]	2019	China	Parallel.	80	40 (50.0%)	65.6 ± 1.8	NR	Berberidaceae; Berberine	NR	4 weeks	Metabolic syndrome	FBS; HOMA-IR; 2hPG
Carvalho et al. [28]	2020	Brazil	Double blind, parallel.	103	71 (69.9%)	58.6 ± 11.1	NR	Zingiberaceae; Ginger	1.2 g	12 weeks	T2 DM	HbA1c; FBS; HOMA-IR
Cheshmeh et al. [29]	2022	Iran	Double blind, parallel.	194	194 (100%)	33.4 ± 5.5	35.0 ± 4.3	Zingiberaceae; Green cardamom	3.0 g	16 weeks	Obese women with PCOS	HbA1c; FBS; Insulin; HOMA-IR; Body weight; BMI
Cicero et al. [30]	2020	Italy	Double blind, parallel.	80	43 (53.8%)	53.5 ± 4.1	27.0 ± 1.8	Zingiberaceae; Curcumin	800 mg	8 weeks	Untreated overweight subjects with suboptimal values of FPG	FBS; Insulin; HOMA-IR; BMI
Dai et al. [31]	2015	China	Double blind, parallel.	69	34 (49.3%)	54.2 ± 11.1	24.3 ± 4.2	Berberidaceae; Berberine	300 mg	96 weeks	Hypertension and T2 DM	HbA1c; FBS
Darmian et al. [32]	2022	Iran	Single blind, parallel.	21	21 (100%)	44.3 ± 2.2	29.2 ± 2.5	Zingiberaceae; Turmeric	2.1 g	8 weeks	Hyperlipidemic T2 DM	FBS; Body weight; BMI
Dastgheib et al. [33]	2022	India	Double blind, parallel.	43	43 (100%)	29.2 ± 5.7	26.9 ± 5.0	Zingiberaceae; Ginger	1.5g	8 weeks	PCOS	FBS; Insulin; HOMA-IR; Body weight; BMI
Derosa et al. [34]	2015	Italy	Double blind, parallel.	85	44 (51.8%)	30.2 ± 7.6	22.74 ± 1.8	Berberidaceae; B. aristata/Silybum marianum	1.18 g BBR + 210 mg SM	24 weeks	T1 DM	HbA1c; FBS; Body weight; BMI; 2hPG
El Gayar et al. [35]	2019	Egypt	Single blind, parallel.	80	39 (48.8%)	46.2 ± 9.1	32.1 ± 1.3	Zingiberaceae; Ginger	1.8 g	8 weeks	T2 DM	HbA1c; FBS; Insulin; HOMA-IR; BMI; 2hPG
Fatemeh et al. [36]	2017	Iran	Double blind, parallel.	80	80 (100%)	47.9 ± 10.3	29.5 ± 3.6	Zingiberaceae; Cardamom	3 g	8 weeks	Overweight and obese prediabetic women	FBS; Insulin; HOMA-IR
Foroutan et al. [37]	2022	Iran	Clinical trial study.	98	55 (51.9%)	60.4 ± 9.4	NR	Zingiberaceae; Ginger	500 mg	12 weeks	T2 DM	HbA1c; FBS; 2hPG
Gao et al. [38]	2013	China	Parallel.	458	288 (62.9%)	50.2 ± 1.6	25.2 ± 0.4	Berberidaceae; Berberine	10 g	36 weeks	Impaired Glucose Tolerance	HbA1c; FBS; BMI; 2hPG
Ghafarzadegan et al. [39]	2021	Iran	Single blind, parallel.	80	26 (32.5%)	54.4 ± 7.0	27.3 ± 3.3	Berberidaceae; B. vulgaris	100 mg	12 weeks	T2 DM	HbA1c; FBS; 2hPG
Guarino et al. [40]	2017	Italy	Double blind, parallel.	136	80 (58.8%)	55.5 ± 8.5	34.0 ± 4.5	Berberidaceae; B. aristata/Silybum marianum	1 g BBR + 210 mg SM	52 weeks	Obese patients with T2 DM	HbA1c; HOMA-IR
Heshmati et al. [41]	2020	Iran	Double blind, parallel.	67	67 (100%)	30.9 ± 6.7	28.0 ± 4.9	Zingiberaceae; Curcumin	1,5 g	12 weeks	PCOS	FBS; Insulin; HOMA-IR; BMI
Hodaei et al. [42]	2019	Iran	Double blind, parallel.	44	22 (50.0%)	59.4 ± 7.4	28.7 ± 3.2	Zingiberaceae; Curcumin	1.5 g	10 weeks	T2 DM	HbA1c; FBS; Insulin; HOMA-IR; Body weight; BMI
Huseini et al. [43]	2023	Iran	Double blind, parallel.	86	54 (62.7%)	54.3 ± 6.0	NR	Zingiberaceae; Turmeric	1 g	12 weeks	T2 DM	HbA1c; FBS
Imani et al. [44]	2015	Iran	Double blind, parallel.	36	15 (41.7%)	56.0 ± 8.1	27.0 ± 3.0	Zingiberaceae; Ginger	1 g	10 weeks	Peritoneal dialysis	FBS; Body weight; BMI
Jiang et al. [45]	2023	China	Double blind, parallel.	31	16 (51.6%)	54.8 ± 10.6	NR	Berberidaceae; Berberine	10 g	12 weeks	T2 DM	HbA1c; FBS; Insulin; HOMA-IR; 2hPG
Karandish et al. [46]	2021	Iran	Double blind, parallel.	41	28 (68.3%)	35.6 ± 7.2	30.7 ± 2.5	Zingiberaceae; Curcumin	400 mg	12 weeks	Overweight or obese prediabeticsubjects	HbA1c; FBS; Insulin; Body weight; BMI; 2hPG
Khajehdehi et al. [47]	2011	Iran	Double blind, parallel.	40	18 (45.0%)	52.8 ± 9.3	NR	Zingiberaceae; Turmeric	1.5 g	8 weeks	Patients with overtype 2 diabetic nephropathy	FBS; 2hPG
Khandouzi et al. [48]	2013	Iran	Double blind, parallel.	41	27 (41.5%)	46.1 ± 7.9	NR	Zingiberaceae; Ginger	2.0 g	12 weeks	T2 DM	HbA1c; FBS
Mahluji et al. [50]	2013	Iran	Double blind, parallel.	58	24 (41.4%)	51.2 ± 6.9	29.5 ± 4.5	Zingiberaceae; Ginger	2 g	8 weeks	T2 DM	HbA1c; FBS; Insulin; HOMA-IR; Body weight; BMI
Mirzabeigi et al. [51]	2015	Iran	Double blind, parallel.	33	9 (27.3%)	62.9 ± 8.5	28.2 ± 3.6	Zingiberaceae; Curcumin	2 g	8 weeks	Patients with coronary artery disease	FBS
Montazerifar et al. [52]	2023	Iran	Double blind, parallel.	40	29 (72.5%)	55.8 ± 9.1	28.5 ± 4.7	Berberidaceae; B. vulgaris	1 g	8 weeks	T2 DM	HbA1c; FBS; Insulin; HOMA-IR; Body weight; BMI
Mozaffari-Khosrav et al. [53]	2014	Iran	Double blind, parallel.	81	50 (61.7%)	50.5 ± 7.5	28.3 ± 5.1	Zingiberaceae; Ginger	3 g	8 weeks	T2 DM	HbA1c; FBS; BMI
Munshi et al. [54]	2023	India	Single blind, parallel.	58	36 (58.1%)	51.5 ± 8.8	28.3 ± 4.4	Zingiberaceae; C. longa	1 g	24 weeks	Prediabetic patients	HbA1c; FBS; Insulin; HOMA-IR; Body weight; BMI; 2hPG
Nematollahi et al. [55]	2022	Iran	Double blind, parallel.	50	34 (68.0%)	27.6 ± 3.7	27.6 ± 3.7	Berberidaceae; Berberine and fenugreek	900 mg BBR + 600 mg fenugreek	12 weeks	T2 DM	HbA1c; FBS; Insulin
Neta et al. [56]	2021	Brazil	Double blind, parallel.	61	47 (77.1%)	62.5 ± 11.0	29.1 ± 4.9	Zingiberaceae; C. longa	500 mg	16 weeks	T2 DM	HbA1c; FBS; Insulin; HOMA-IR; BMI
Nieman et al. [57]	2012	USA	Double blind, parallel.	30	30 (100%)	55.7 ± 7.7	NR	Zingiberaceae; Turmeric	2.8 g	4 weeks	Overweight patients	FBS; Body weight
Panahi et al. [58]	2018	Iran	Double blind, parallel.	100	49 (49.0%)	42.0 ± 7.6	25.6 ± 2.1	Zingiberaceae; Curcumin	500 mg	12 weeks	T2 DM	HbA1c; FBS; Insulin; HOMA-IR; Body weight; BMI
Panigrahi et al. [59]	2023	India	Double blind, parallel.	34	26 (76.5%)	44.2 ± 8.6	25.5 ± 4.2	Berberidaceae; Berberine	1.5 g	12 weeks	Prediabetic patients	HbA1c; FBS; Insulin; HOMA-IR; 2hPG
Rafie et al. [60]	2020	Iran	Double blind, parallel.	46	26 (56.6%)	49.0 ± 9.7	31.3 ± 3.0	Zingiberaceae; Ginger	1.5 g	12 weeks	Patientswith NAFLD	FBS; Insulin; HOMA-IR; Body weight; BMI
Rahimi et al. [61]	2016	Iran	Double blind, parallel.	70	39 (55.7%)	58.7 ± 11.1	27.1 ± 3.2	Zingiberaceae; NanoCurcumin	80 mg	12 weeks	T2 DM	HbA1c; FBS; BMI
Rahimlou et al. [62]	2019	Iran	Double blind, parallel.	37	16 (43.2%)	43.9 ± 8.4	30.5 ± 2.7	Zingiberaceae; Ginger	2 g	12 weeks	Patients with metabolicsyndrome	FBS; HOMA-IR; Body weight; BMI
Rahmani et al. [63]	2016	Iran	Double blind, parallel.	77	42 (52.5%)	47.7 ± 10.7	31.1 ± 5.1	Zingiberaceae; Curcumin	500 mg	8 weeks	NAFLD	HbA1c; FBS; Body weight; BMI
Rashidi et al. [64]	2018	Iran	Double blind, parallel.	81	50 (61.7%)	47.6 ± 7.8	29.5 ± 7.3	Berberidaceae; Berberine	1 g	4 weeks	T2 DM	FBS; Insulin; HOMA-IR; BMI; 2hPG
Rondanelli et al. [65]	2023	Italy	Double blind, parallel.	49	28 (57.1%)	59.5 ± 7.8	29.8 ± 3.3	Berberidaceae; Berberine	550 mg	8 weeks	Overweight patients with an IFG sta-tus	HbA1c; FBS; Insulin; HOMA-IR
Sartore et al. [66]	2021	Italy	Single blind, parallel.	40	8 (20.0%)	67.1 ± 6.1	28.9 ± 4.7	Berberidaceae; B. aristata	250 mg	12 weeks	T2 DM	FBS; Insulin; HOMA-IR; BMI
Sharma et al. (a) [67]	2017	India	Parallel, absence of blinding.	60	NR	NR	NR	Berberidaceae; B. aristata	1.5 g	36 weeks	T2 DM	FBS
Sharma et al. (b) [67]	2017	India	Parallel, absence of blinding.	60	NR	NR	NR	Berberidaceae; B. aristata	3 g	36 weeks	T2 DM	FBS
Shidfar et al. [69]	2012	Iran	Double blind, parallel.	42	NR	52.7 ± 5.6	27.5 ± 1.0	Berberidaceae; Barberry	3 g	12 weeks	T2 DM	HbA1c; FBS; Insulin; HOMA-IR; Body weight; BMI
Shidfar et al. [68]	2015	Iran	Double blind, parallel.	45	NR	46.2 ± 8.0	29.4 ± 2.8	Zingiberaceae; Ginger	3 g	12 weeks	T2 DM	HbA1c; FBS; Insulin; HOMA-IR; Body weight; BMI
Sohaei et al. [70]	2019	Iran	Double blind, parallel.	51	51 (100%)	29.6 ± 5.1	30.5 ± 4.2	Zingiberaceae; Curcumin	1 g	6 weeks	PCOS	FBS; Insulin; HOMA-IR
Sousa et al. [71]	2020	Brazil	Double blind, parallel.	61	47 (77.1%)	62.6 ± 11.0	29.3 ± 4.9	Zingiberaceae; Long turmeric	500 mg	16 weeks	T2 DM	HbA1c; FBS; Insulin; HOMA-IR; Body weight; BMI;
Tahmasebi et al. [72]	2019	Iran	Double blind, parallel.	80	48 (60.0%)	53.6 ± 7.8	NR	Berberidaceae; B. vulgaris	1g	6 weeks	T2 DM	FBS; 2hPG
Zakerkish et al. [73]	2023	Iran	Double blind, parallel.	60	40 (66.7%)	55.2 ± 9.8	28.8 ± 4.7	Zingiberaceae; Turmeric + Ginger + Black pepper	948 mg	12 weeks	T2 DM	HbA1c; FBS; Insulin; HOMA-IR; BMI
Zhang et al. [5]	2008	China	Double blind, parallel.	106	49 (44.5%)	51 ± 9.4	25.5 ± 3.5	Berberidaceae; Berberine	1 g	12 weeks	Dyslipidemia and T2 DM	HbA1c; FBS; Insulin; HOMA-IR; Body weight; BMI; 2hPG

2hPG—2-h, plasma, glucose; BMI—body, mass, index; FBS—fasting, blood, sugar; HbA1c—glycated, hemoglobin; HOMA-IR—homeostatic, model, assessment, of, insulin, resistance; IFG—impaired, fasting, glucose.

## Data Availability

Data are contained within the article.

## Supplementary Materials

The following supporting information can be downloaded at: https://www.mdpi.com/article/10.3390/ijms26125565/s1.

## Abbreviations

The following abbreviations are used in this manuscript:

AMPK	AMP-Activated Protein Kinase
BBR	Berberine
BMI	Body Mass Index
FBG	Fasting Blood Glucose
FBS	Fasting Blood Sugar
GLUT-4	Glucose Transporter Type 4
HbA1c	Hemoglobin A1c
HOMA-IR	Homeostasis Model Assessment of Insulin Resistance
IFG	Impaired Fasting Glucose
NAFLD	Non-Alcoholic Fatty Liver Disease
NF-κB	Nuclear Factor Kappa B
PCOS	Polycystic Ovary Syndrome
PEPCK	Phosphoenolpyruvate Carboxykinase
PICOS	Population, Intervention, Comparison, Outcome, Study Design
PRISMA	Preferred Reporting Items for Systematic Reviews and Meta-Analyses
PROSPERO	International Prospective Register of Systematic Reviews
RCT	Randomized Controlled Trial
SCFAs	Short-Chain Fatty Acids
T2 DM	Type 2 Diabetes Mellitus
WHR	Waist-to-Hip Ratio
2hPG	2-Hour Postprandial Glucose
