# Supplementary figures and images for: Effect of the Consumption of Species from the Zingiberaceae or Berberidaceae Family on Glycemic Profile Parameters: A Systematic Review and Meta-Analysis
Source: Int J Mol Sci. 2025 Jun 10;26(12):5565. doi: 10.3390/ijms26125565 (PMC12192924; doi:10.3390/ijms26125565)

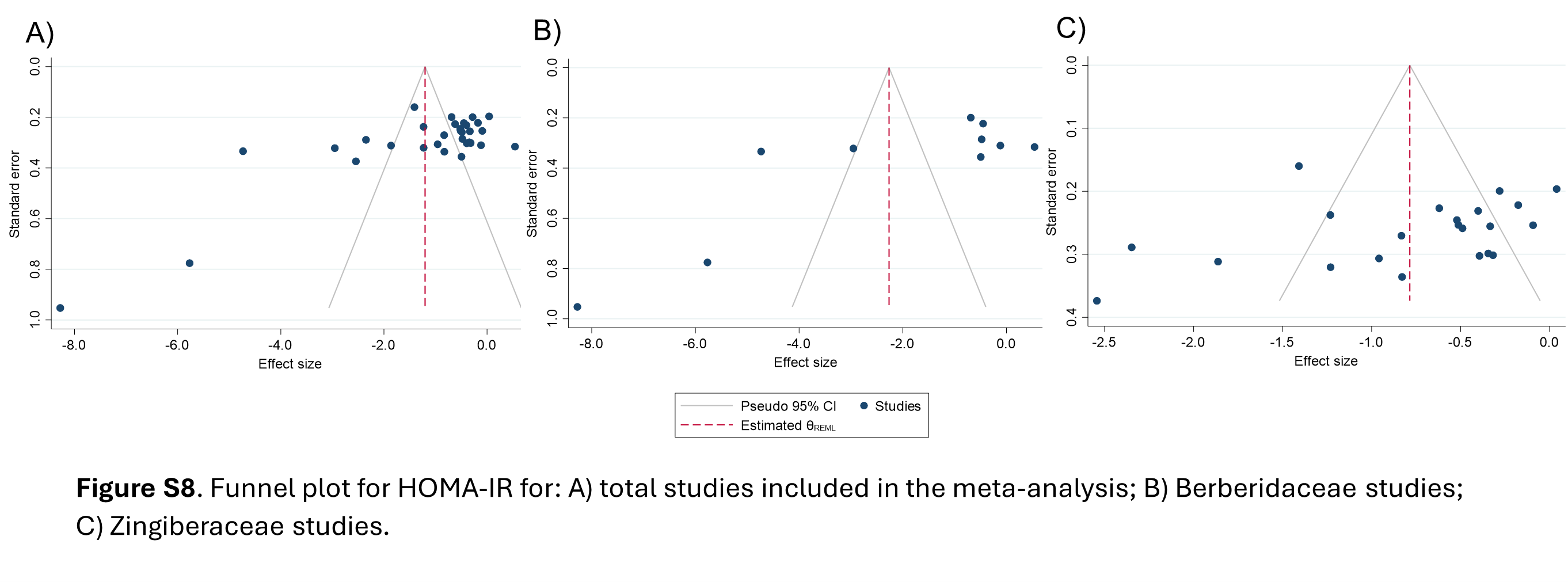

Supplement: Supplementary file 1 [file ijms-26-05565-s001.zip › Figures Supplementary Material/Figure S8.tif]

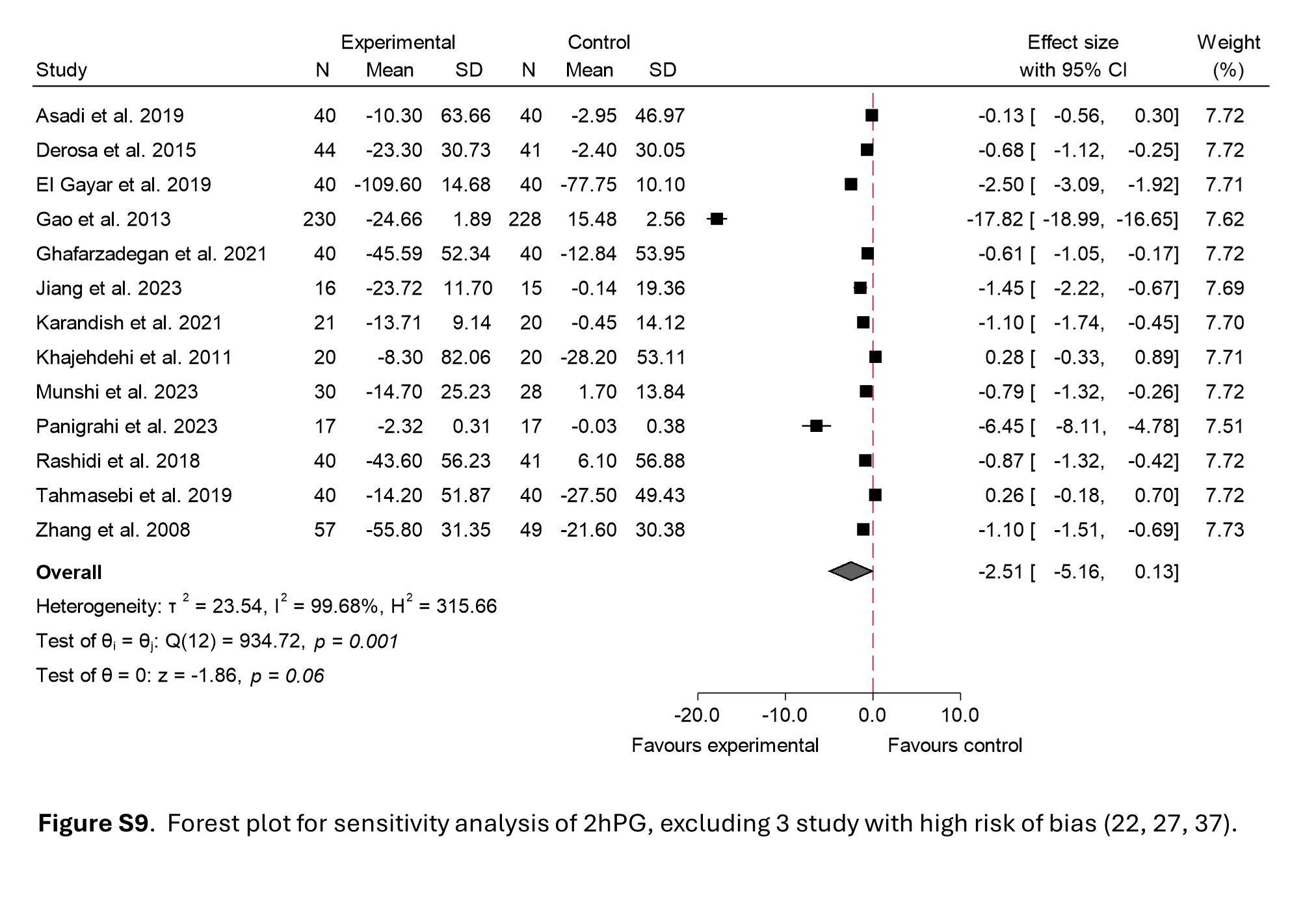

Supplement: Supplementary file 1 [file ijms-26-05565-s001.zip › Figures Supplementary Material/Figure S9.tif]

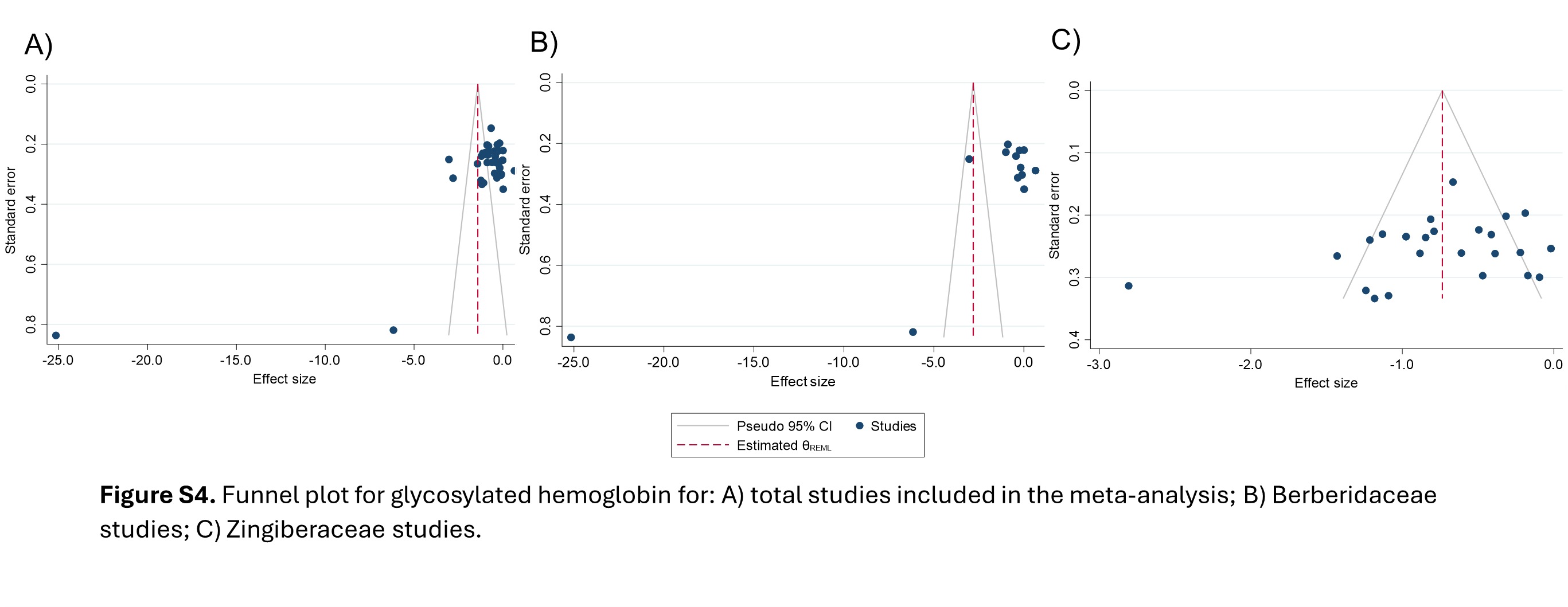

Supplement: Supplementary file 1 [file ijms-26-05565-s001.zip › Figures Supplementary Material/Figure S4.tif]

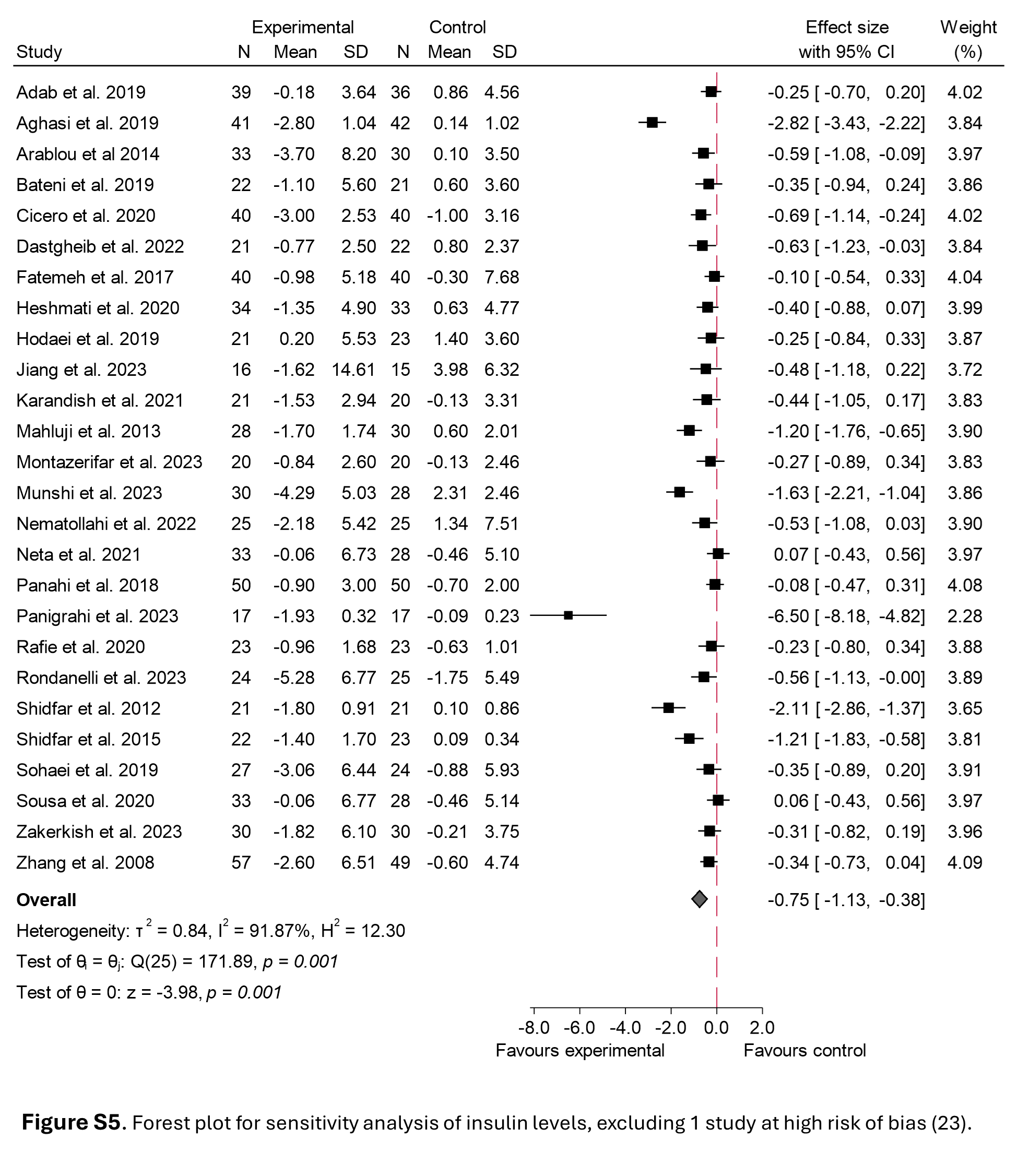

Supplement: Supplementary file 1 [file ijms-26-05565-s001.zip › Figures Supplementary Material/Figure S5.tif]

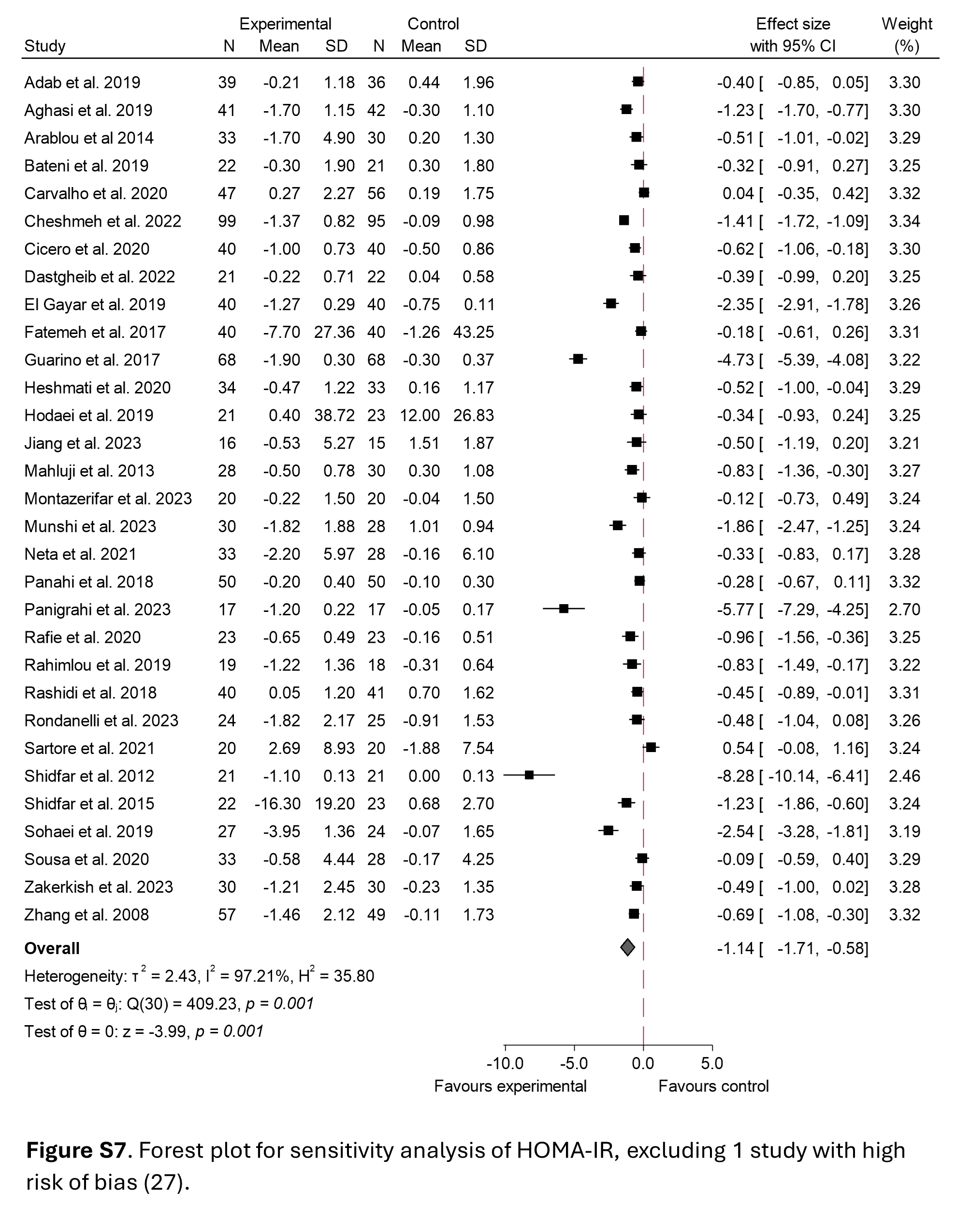

Supplement: Supplementary file 1 [file ijms-26-05565-s001.zip › Figures Supplementary Material/Figure S7.tif]

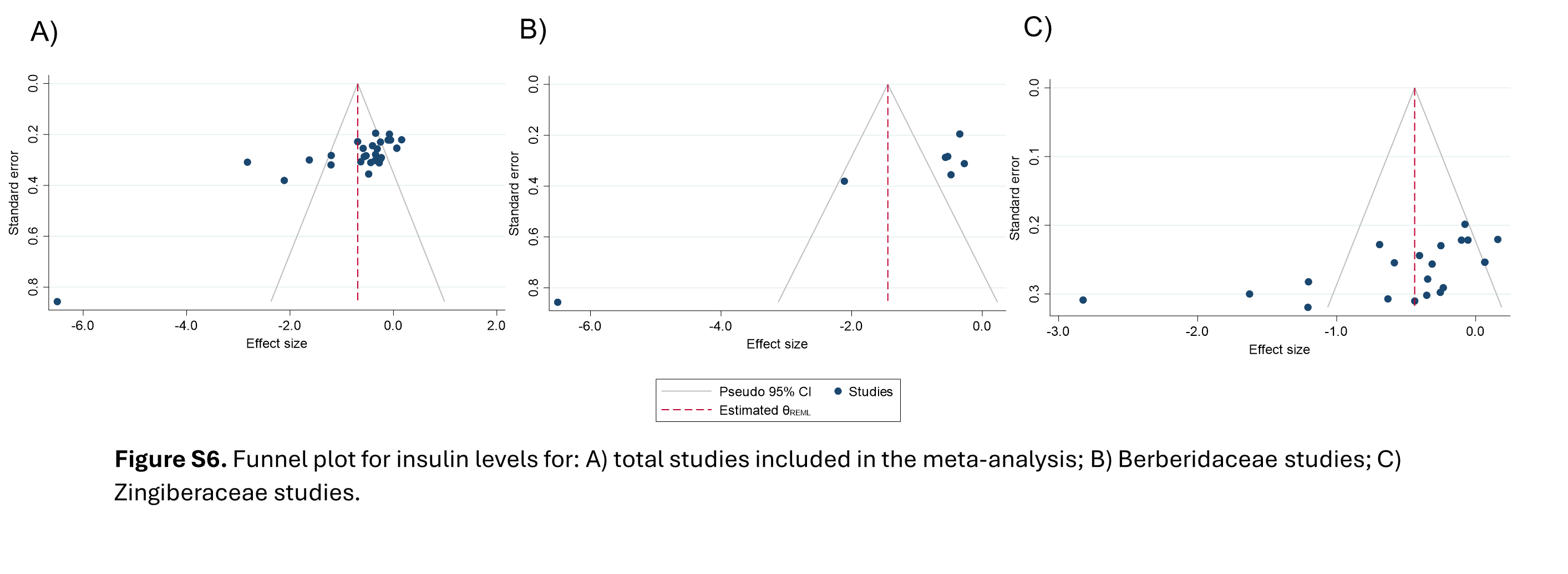

Supplement: Supplementary file 1 [file ijms-26-05565-s001.zip › Figures Supplementary Material/Figure S6.tif]

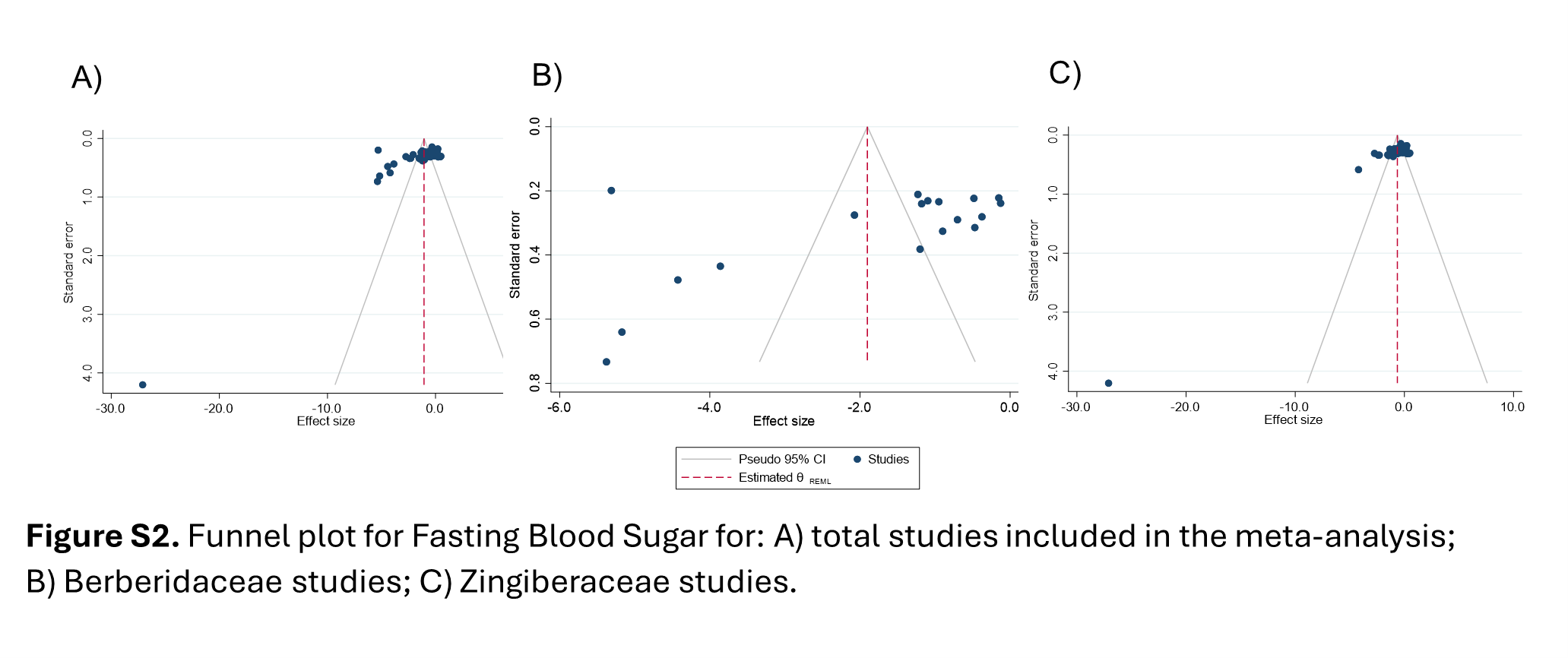

Supplement: Supplementary file 1 [file ijms-26-05565-s001.zip › Figures Supplementary Material/Figure S2.tif]

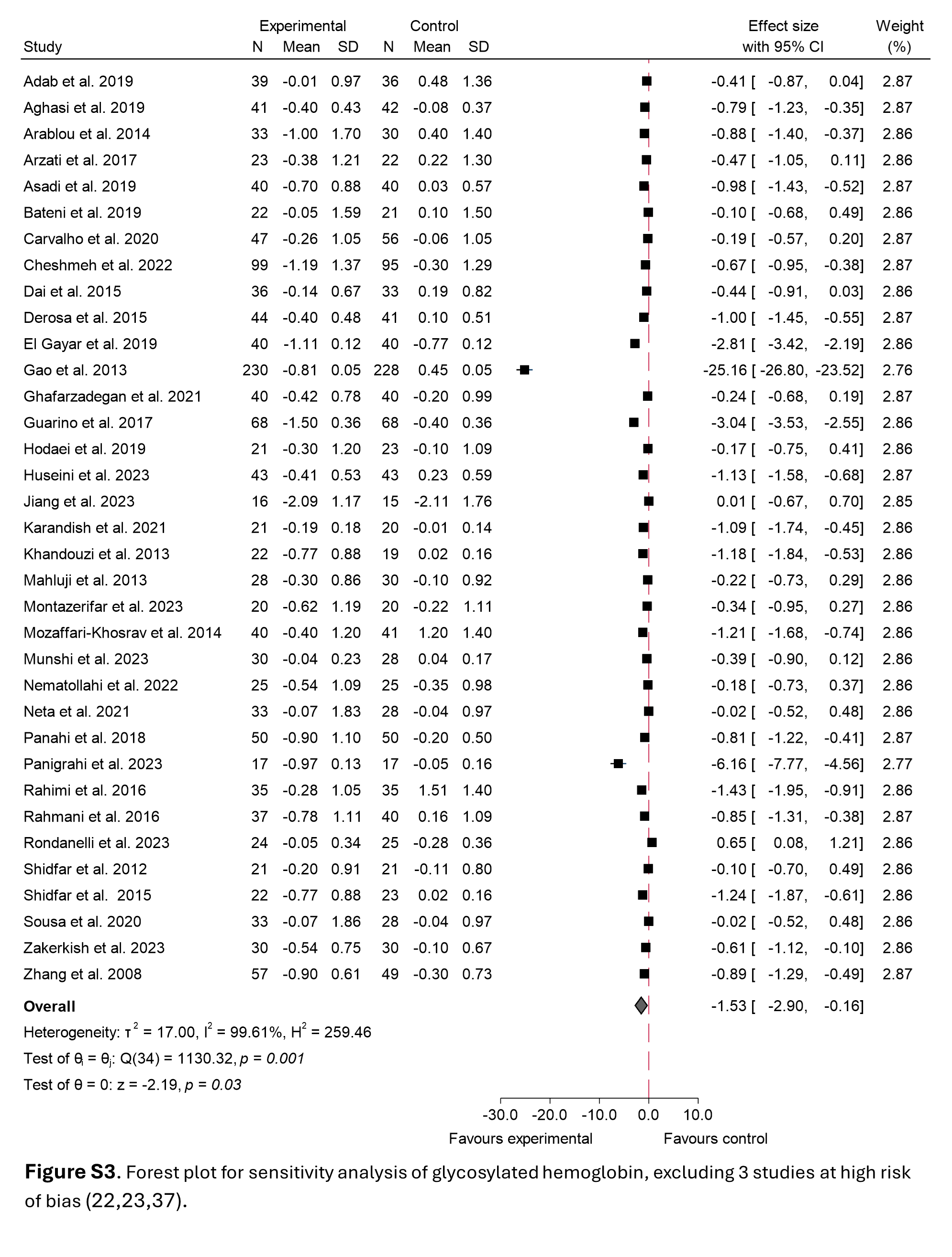

Supplement: Supplementary file 1 [file ijms-26-05565-s001.zip › Figures Supplementary Material/Figure S3.tif]

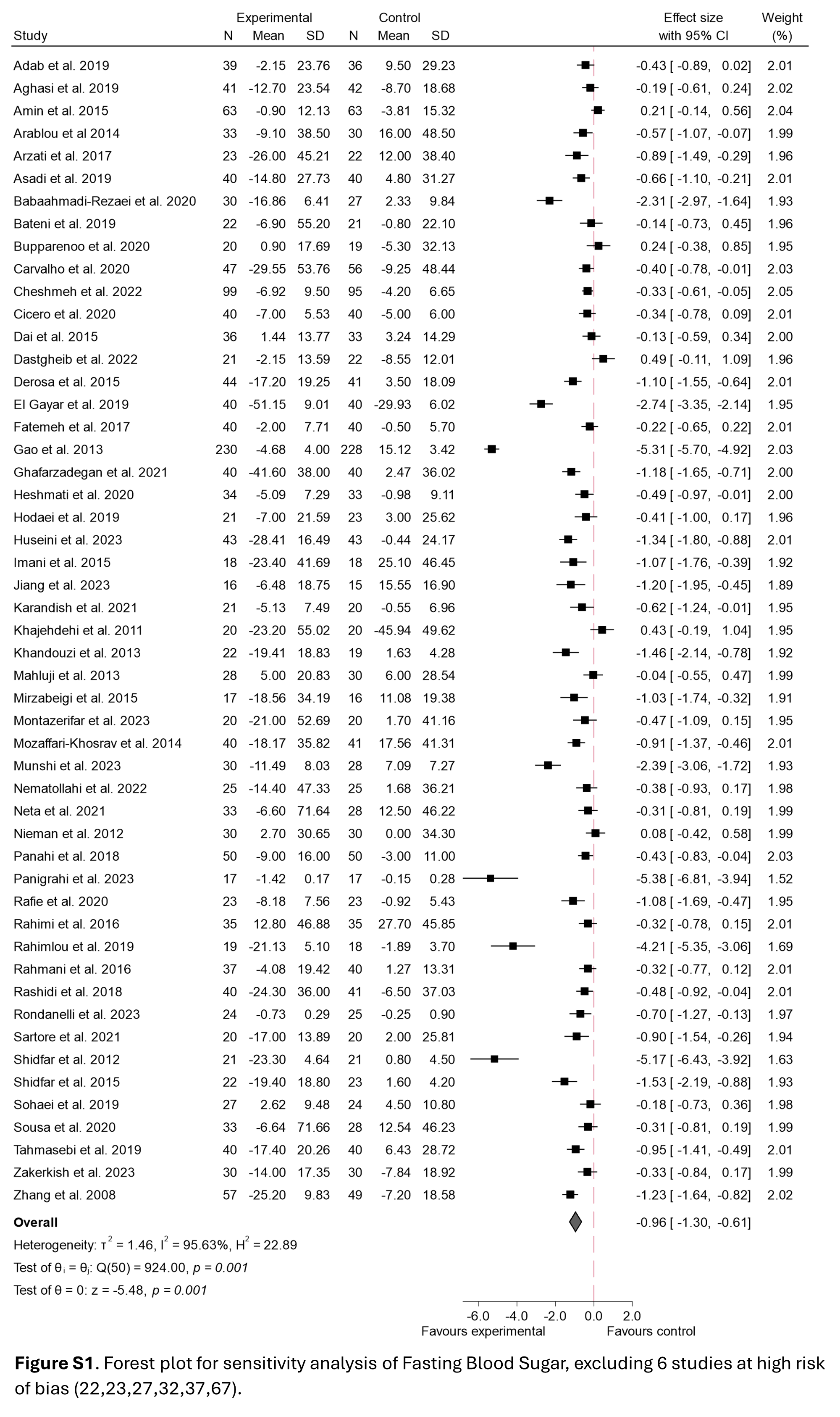

Supplement: Supplementary file 1 [file ijms-26-05565-s001.zip › Figures Supplementary Material/Figure S1.tif]

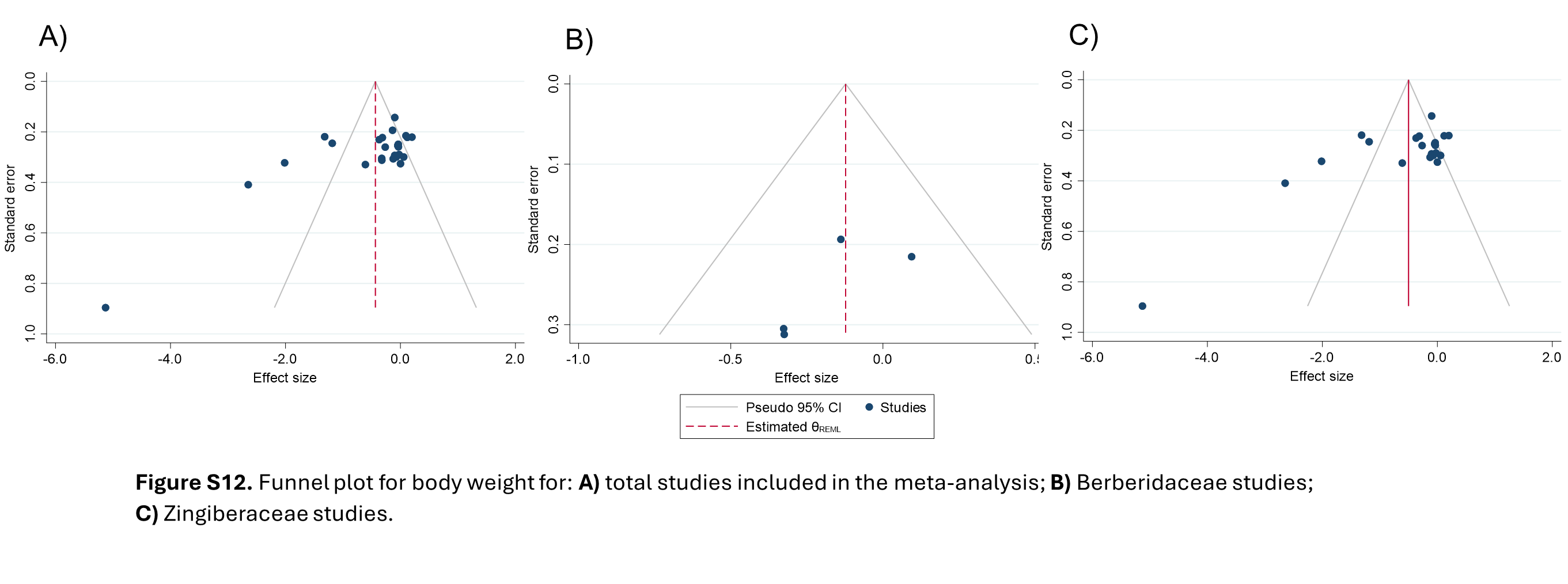

Supplement: Supplementary file 1 [file ijms-26-05565-s001.zip › Figures Supplementary Material/Figure S12.tif]

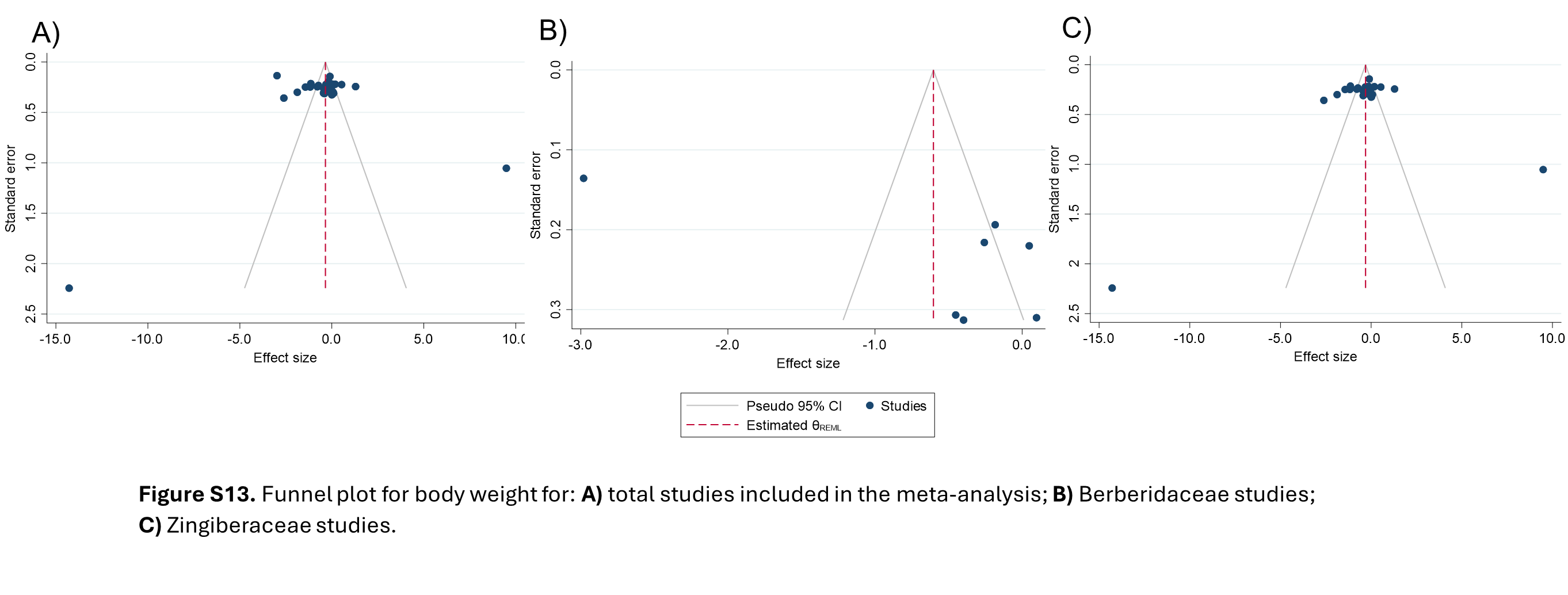

Supplement: Supplementary file 1 [file ijms-26-05565-s001.zip › Figures Supplementary Material/Figure S13.tif]

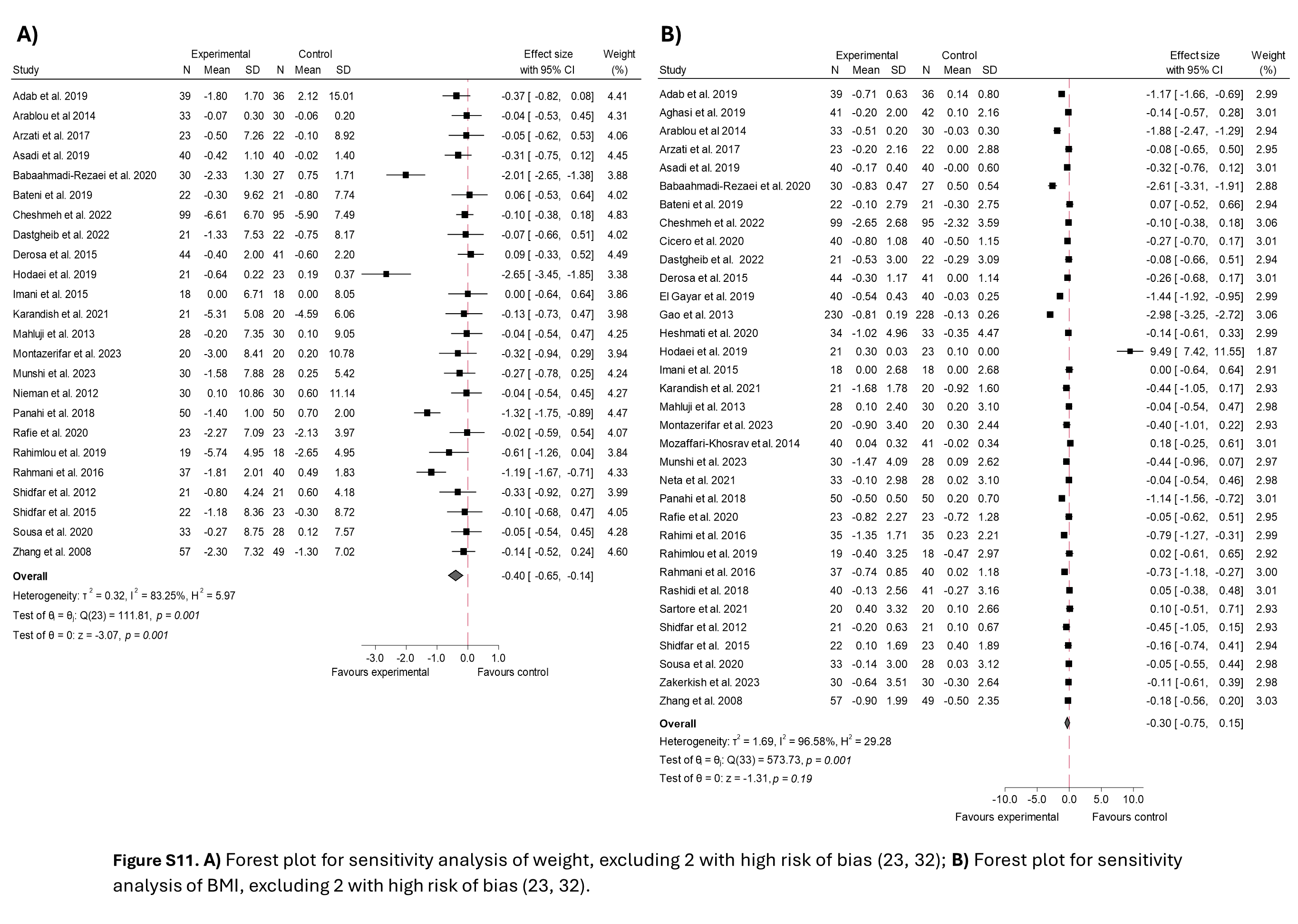

Supplement: Supplementary file 1 [file ijms-26-05565-s001.zip › Figures Supplementary Material/Figure S11.tif]

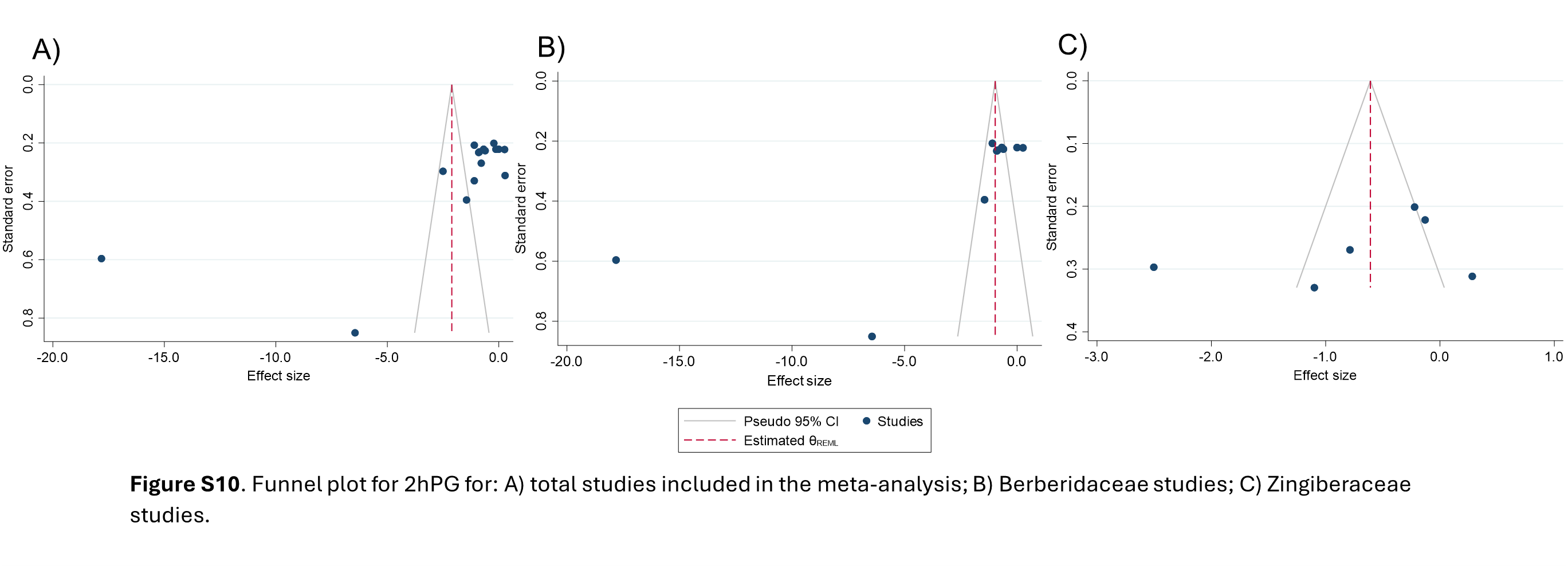

Supplement: Supplementary file 1 [file ijms-26-05565-s001.zip › Figures Supplementary Material/Figure S10.tif]
